# The position of nonsense mutations can predict the phenotype severity: A survey on the DMD gene

**DOI:** 10.1371/journal.pone.0237803

**Published:** 2020-08-19

**Authors:** Annalaura Torella, Mariateresa Zanobio, Roberta Zeuli, Francesca del Vecchio Blanco, Marco Savarese, Teresa Giugliano, Arcomaria Garofalo, Giulio Piluso, Luisa Politano, Vincenzo Nigro

**Affiliations:** 1 Dipartimento di Medicina di Precisione, Università degli Studi della Campania “Luigi Vanvitelli”, Napoli, Italy; 2 Telethon Institute of Genetics and Medicine (TIGEM), Pozzuoli, Italy; 3 Folkhälsan Research Center, Medicum, University of Helsinki, Helsinki, Finland; 4 Dipartimento di Medicina Sperimentale, Università degli Studi della Campania “Luigi Vanvitelli”, Napoli, Italy; Iowa State University, UNITED STATES

## Abstract

A nonsense mutation adds a premature stop signal that hinders any further translation of a protein-coding gene, usually resulting in a null allele. To investigate the possible exceptions, we used the *DMD* gene as an ideal model. First, because dystrophin absence causes Duchenne muscular dystrophy (DMD), while its reduction causes Becker muscular dystrophy (BMD). Second, the *DMD* gene is X-linked and there is no second allele that can interfere in males. Third, databases are accumulating reports on many mutations and phenotypic data. Finally, because *DMD* mutations may have important therapeutic implications. For our study, we analyzed large databases (LOVD, HGMD and ClinVar) and literature and revised critically all data, together with data from our internal patients. We totally collected 2593 patients. Positioning these mutations along the dystrophin transcript, we observed a nonrandom distribution of BMD-associated mutations within selected exons and concluded that the position can be predictive of the phenotype. Nonsense mutations always cause DMD when occurring at any point in fifty-one exons. In the remaining exons, we found milder BMD cases due to early 5’ nonsense mutations, if reinitiation can occur, or due to late 3’ nonsense when the shortened product retains functionality. In the central part of the gene, all mutations in some in-frame exons, such as in exons 25, 31, 37 and 38 cause BMD, while mutations in exons 30, 32, 34 and 36 cause DMD. This may have important implication in predicting the natural history and the efficacy of therapeutic use of drug-stimulated translational readthrough of premature termination codons, also considering the action of internal natural rescuers. More in general, our survey confirm that a nonsense mutation should be not necessarily classified as a null allele and this should be considered in genetic counselling.

## Introduction

A nonsense mutation is classically considered a loss-of-function change, with ribosomes that dissociate from mRNA and transcript degradation. Shortened protein products are usually quickly ubiquitinated and digested by the proteasome. All these mechanisms must be very efficient to prevent cell accumulation of toxic or ectopic protein garbage [1]. This suggests that the functional effect of a nonsense mutation may be considered equivalent to the full deletion of a gene (null or amorph allele). However, the difference between the two causes is striking: in the case of a nonsense mutation the cell retains almost all the genetic information, while in the case of deletion does not. We searched for exceptions by studying nonsense mutations of the *DMD* gene encoding a 427kDa- protein, named dystrophin. This is an ideal model. First, because DMD is X-linked and in hemizygous males there is no second allele that may complicate the genotype/phenotype correlation. Second, because in males the null alleles are fully penetrant in the form of Duchenne muscular dystrophy (DMD) and well distinct from the hypomorphic alleles that cause Becker muscular dystrophy (BMD). Third, because a huge number of different nonsense mutations and phenotypic data have been reported since 1992. Finally, because *DMD* nonsense mutations are the target for treatments based on readthrough strategies [2, 3]. Dystrophin defects disrupt the associated glycoprotein complex at the sarcolemma and several pathogenic cascades are thus activated [4]. They quickly lead to structural and functional disruption of the muscles and to a progressive muscle weakness. DMD is the most severe phenotype, in which the progressive muscle disruption cause an early loss of ambulation, skeletal alterations with respiratory and cardiac involvement, and sometimes cognitive impairment [5]. Conversely, BMD represents the milder phenotype with a slower progression of muscle weakness, tardive loss of ambulation, and variable cardiac and respiratory involvement [6]. DMD diagnosis cannot be questioned in teenagers, considering the dramatic phenotype in males, such as difficulty running, climbing stairs, getting up from the floor with a positive Gowers maneuver, creatine kinase values up to 100 times the normal maximum value, and the high accuracy of natural history data available. Even if phenotype variants have been reported, these are never strong enough to associate the dystrophin absence to a BMD phenotype. Another point regards clinical trials, because any phenotype variability in patients with nonsense mutations may reduce the statistical significance of any therapeutic improvement [7, 8].

The full mutational analysis of the *DMD* gene is considered part of the standard of care for DMD. The *DMD* gene, consisting of 79 exons generally separated by huge introns, is prone to intragenic deletions or duplications that when include exons cause DMD or BMD [9–11]. The first nonsense variants and other small defects were only identified six years after the *DMD* gene cloning [12, 13]. Unlike most disease genes, single nucleotide substitutions and small insertion/deletion of bases are a less frequent cause of disease [14, 15]. Random nonsense mutations were found in 10–15% of DMD cases [16]. This randomness of lethal X-linked mutations confirms the Haldane’s rule and offers a possibility of unbiased analysis [17]. In 1996, a pivotal study paved the way for a new therapeutic option for genetic disorders caused by nonsense alleles: gentamycin was shown to induce the readthrough of ribosome overcoming a single stop codon in the context of an open reading frame in the cystic fibrosis gene [18]. However, any possible therapeutic window was closed by severe side effects of gentamycin. A high-throughput screening of synthetic molecules resulted in the selection of a new compound, named PTC-124 from PTC Therapeutics (New Jersey, USA) that showed an important increase of protein production in cells and *mdx* mice, carrying a nonsense variant in exon 23 [2, 19, 20]. This drug, commercial name Ataluren (Translarna), can be administered orally and, compared with aminoglycosides, shows fewer side effects, in about 5% of treated subjects. These include vomiting, diarrhea, nausea (feeling sick), headache, stomachache and flatulence [21, 22]. Despite weak Phase II results, its use was approved in member states of the European Union, Iceland, Israel, Kazakhstan, Liechtenstein, Norway and the Republic of Korea, for the treatment (40 mg/kg/day) of ≥ 2 years DMD boys caused by nonsense mutation, or aged ≥ 5 years in Brazil and Chile [3, 23]. The possibility of readthrough-based treatments provided further impetus in searching for nonsense mutations in DMD boys as early as possible. Nowadays, next generation sequencing (NGS) protocols are being applied to fully sequence DNA in children with suspected muscular dystrophy [24–27].

Our present survey on the positional effect of nonsense mutations may have important implication for therapeutic use of drug-stimulated translational readthrough of premature termination codons.

## Methods

We collected the published unique nonsense variants in the dystrophin gene (*DMD*, NM_004006.2) from three main databases: Leiden Open Variation Database (LOVD) [28], Human Genome Variant Database (HGMD) [29], and ClinVar [30]. Data filtering was based on their classification as “Pathogenic” variants and considering their molecular consequence differently termed in the three databases (by using HGVS nomenclature in LOVD, “Term” in HGMD, and “nonsense” in ClinVar). We selected 702 nonsense variants in LOVD, 823 in HGMD and 236 in ClinVar. Removing the duplicates among the databases and integrating all the data, we obtained 849 unique nonsense mutations so far published (until April 2020). LOVD also provides a rough indication of the variant recurrence, as it allows researcher to resubmit a known variant found in additional patients [31]. Literature data were used to carefully correlate the specific phenotype to the nonsense variant observed in each patient. We reviewed these data together with our internal cohort of 1,102 patients that included already published cases [13, 15, 27] and further 128 cases. Genomic DNA was extracted from leucocyte according to the standard procedure [32]. We performed Multiplex Ligation-dependent Probe Amplification (MLPA), according to the manufacturer’s recommendations (MRC Holland) and/or Log-PCR, as previously described [33]. MLPA/LogPCR negative patients were analyzed for single nucleotide variants or small ins/del performing the NGS MotorPlex panel [26, 27] or by a panel focused on >5,200 genes responsible for Mendelian Disease (Sure Select Agilent Custom Constitutional Panel). We also used Human Splice Finder (HSF) [34], a bioinformatic tool able to predict possible effects of the mutations on canonical or cryptic splice sites and on specific exonic splicing enhancer/silencer sequences (ESE/ESS) [35]. ProteinPaint [36] was used to graphically represent the distribution of nonsense mutations along *DMD* gene. The Ethics Committee of Vanvitelli University approved the study with ID 5586/19 and 8635/19.

## Results and discussion

To search for the most comprehensive number of annotated nonsense mutations in the *DMD* gene, we added to our internal cases all the variants retrieved from public databases (LOVD [28], HGMD [29] and ClinVar [30]) or from literature. The largest published study was carried out on 243 patients with nonsense mutations by Flanigan et al [37], but all recent papers were also considered [38, 39]. Since in some cases, nonsense variants reported in public databases did not have a clear clinical diagnosis, we critically reviewed the associated reports to be sure of the assigned phenotype. In our patient cohort, we had accurate information on 61 cases with nonsense mutations in the DMD gene, part of which was previously published (S1 Table) [13, 15, 40]. Altogether, we collected 2593 patients with 849 unique nonsense mutations (S2 Table). The reports were classified in five groups based on the phenotypic annotation of the patients: DMD, BMD, DMD/BMD, ND (Not Defined) and Other, as showed in Table 1.

**Table 1:** Summary of nonsense mutations and patients classified on the basis of reported phenotypes.

Disease	Unique nonsense mutations	Number of patients
**DMD**	579	2022
**BMD**	54	180
**DMD/BMD**	104	245
**ND**	88	103
OTHER^*^	25	43
**TOTAL**	849	2593

*This category includes heterozygous symptomatic carrier, hyperCK, or cardiomyopathic phenotypes.

**Fig 1:**
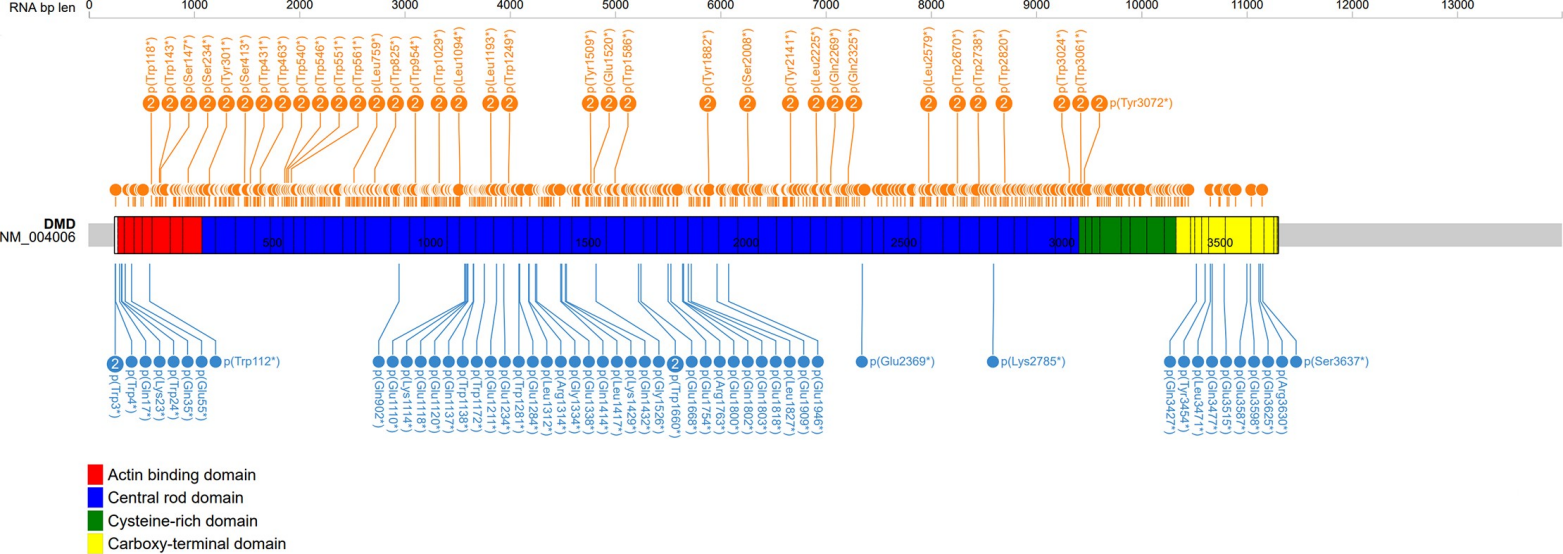
Graphical representation of the distribution of nonsense mutations in the DMD gene. Nonsense mutations associated with DMD (orange) or BMD (blue) are reported. The ProteinPaint graph [36] highlights three main regions for BMD phenotypes, while the vast majority of nonsense mutations are associated with DMD. The number in the circle indicates that different nucleotide changes determine the same nonsense codon.

**Fig 2:**
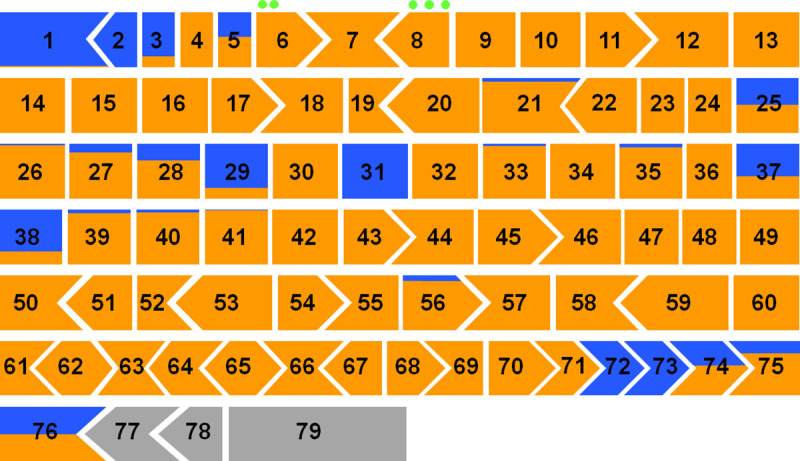
Color representation of the distribution of phenotypes in relation to *DMD* exons.

To evaluate any positional effect of nonsense mutations, we first considered their distribution along the *DMD* gene in association with different phenotypes (Fig 1). Three main BMD-associated coding regions are evident: N-terminus, C- terminus and central part of the rod domain. BMD-associated nonsense mutations are listed in Table 2. Fig 2 describes the percentage of DMD/BMD frequency for each DMD exon with a blue color code for BMD cases and orange for DMD cases. From the analysis of this figure, we immediately observed a non-random distribution of milder cases in specific exons. We also found that mutations in adjacent exons, in the middle part of the gene, had completely different phenotypic consequences. For example, mutations in exons 30 and 32 were DMD-linked, while mutations in exons 29 and 31 were BMD-linked. We identified four exons with 15 unique nonsense mutations never associated with DMD. These were exons 2, 31, 72 and 73, for which we only found BMD patients or milder phenotypes. In addition, we highlighted other 21 exons with 236 nonsense mutations associated with BMD, DMD phenotype or undefined phenotypes at different frequencies (Table 3). Finally, nonsense mutation in the remaining 51 exons are associated with DMD in 100% of cases, as originally expected for “loss-of-function” mutations [41].

**Table 2:** Summary of reported nonsense mutations associated to BMD/mild phenotype.

EXON	CDS	Protein	hg19	Patients	Effect	Reference
1	c.8G>A	p.(Trp3*)	g.33229422C>T	1	-	[28]
1	c.9G>A	p.[Trp3*, Leu2_Met124del, Leu2_Met128del]	g.33229421C>T	27	exon 2-3-4-5 skipping	[38]
1	c.11G>A	p.(Trp4*)	g.33229419C>T	6	-	[42]
2	c.49C>T	p.(Gln17*)	g.33038300G>A	1	-	[39]
2	c.67A>T	p.(Lys23*)	g.33038282T>A	1	-	[28]
2	c.72G>A	p.(Trp24*)	g.33038277C>T	1	-	[43]
3	c.103C>T	p.(Gln35*)	g.32867928G>A	2	-	[44]
3	c.163G>T	p.[Glu55*, Phe32Metfs*13]	g.32867868C>A	1	frame-shift deletion of exons 3–7	[45]
5	c.336G>A	p.(Trp112*)	g.32841433C>T	3	-	[42]
21	c.2704C>T	p.(Gln902*)	g.32503135G>A	2	-	[46]
25	c.3328G>T	p.[Glu1110*, Leu1093_Gln1144del]	g.32481660C>A	1	exon 25 skipping	[45]
25	c.3340A>T	p.(Lys1114*)	g.32481648T>A	3	-	[8, 9]
25	c.3352G>T	p.(Glu1118*)	g.32481636C>A	2	-	[47]
25	c.3358G>T	p.(Glu1120*)	g.32481630C>A	1	-	[48]
25	c.3409C>T	p.(Gln1137*)	g.32481579G>A	3	-	[49]
25	c.3413G>A	p.(Trp1138*)	g.32481575C>T	4	-	[50]
26	c.3515G>A	p.[Trp1172*, Val1145_Lys1201del]	g.32472867C>T	1	exon 26 skipping	[51, 52]
27	c.3631G>T	p.[Glu1211*; Arg1202_1262del; Arg1202_1357del]	g.32466728C>A	1	exons 27 or 27-28-29 skipping	[53, 54]
27	c.3700G>T	p.(Glu1234*)	g.32466659C>A	1	-	[9]
28	c.3843G>A	p.(Trp1281*)	g.32459375C>T	2	-	[55]
28	c.3850G>T	p.[Glu1284*, Glu1263_Asp1307del, Glu1263_Glu1357del]	g.32459368C>A	2	exon 28 or 28–29 skipping	[42]
29	c.3935T>A	p.[Leu1312*, Glu1263_Glu1357del, Ser1308_Glu1357del]	g.32456494A>T	1	exon 29 or 28–29 skipping	[28, 56]
29	c.3940C>T	p.[Arg1314*, Glu1263_Glu1357del, Ser1308_Glu1357del]	g.32456489G>A	31	exon 29 or 28–29 skipping	[51, 56]
29	c.4000G>T	p.[Gly1334*, Glu1263_Glu1357del, Ser1308_Glu1357del]	g.32456429C>A	1	exon 29 or 28–29 skipping	[28, 56]
29	c.4012G>T	p.(Glu1338*)	g.32456417C>A	1	-	[9]
31	c.4240C>T	p.(Gln1414*)	g.32408292G>A	1	-	[9]
31	c.4250T>A	p.[Leu1417*; Ile1413_Lys1449del]	g.32408282A>T	4	exon 31 skipping	[57]
31	c.4285A>T	p.(Lys1429*)	g.32408247T>A	1	-	[9]
31	c.4294C>T	p.[Gln1432*, Ile1413_Lys1449del]	g.32408238G>A	3	exon 31 skipping	[58]
33	c.4576G>T	p.(Gly1526*)	g.32404525C>A	1	-	[28]
35	c.4979G>A	p.(Trp1660*)	g.32383183C>T	1	-	[15]
35	c.4980G>A	p.(Trp1660*)	g.32383182C>T	1	-	[28]
35	c.5002G>T	p.(Glu1668*)	g.32383160C>A	1	-	[59]
37	c.5260G>T	p.(Glu1754*)	g.32380970C>A	1	-	[60]
37	c.5287C>T	p.[Arg1763*, Arg1719_Lys1775del]	g.32380943G>A	22	exon 37 skipping	[61]
38	c.5398G>T	p.(Glu1800*)	g.32366573C>A	3	-	[9]
38	c.5404C>T	p.(Gln1802*)	g.32366567G>A	5	-	[62]
38	c.5407C>T	p.[Gln1803*, Ala1776_Met1816del]	g.32366564G>A	5	exon 38 skipping	[46, 56]
39	c.5452G>T	p.(Glu1818*)	g.32364194C>A	1	-	[28]
39	c.5480T>A	p.[Leu1827*, Ala1776_Lys1862del, Asn1817_Lys1862del]	g.32364166A>T	2	exons 38–39 skipping	[28, 56]
40	c.5725G>T	p.(Glu1909*)	g.32361265C>A	1	-	[9]
41	c.5835G>T	p.(Glu1946*)	g.32360304C>A	1	-	[28]
49	c.7105G>T	p.[Glu2369*, Glu2367_Lys2400del]	g.31854930C>A	5	exon 49 skipping	[61]
56	c.8353A>T	p.(Lys2785*)	g.31525435T>A	1	-	[9]
72	c.10279C>T	p.[Gln3427*, Pro3422_Arg3443del]	g.31191705G>A	7	exon 72 skipping	[61]
73	c.10362T>A	p.(Tyr3454*)	g.31190497A>T	1	-	[28]
74	c.10412T>A	p.(Leu3471*)	g.31187701A>T	2	-	[57]
74	c.10429C>T	p.(Gln3477*)	g.31187684G>A	1	-	[28]
74	c.10543G>T	p.[Glu3515*, Ile3465_Arg3518delinsMet]	g.31187570C>A	1	exon 74 skipping	[52]
75	c.10759G>T	p.(Glu3587*)	g.31165430C>A	1	-	[28]
75	c.10792G>T	p.(Glu3598*)	g.31165397C>A	1	-	[46]
76	c.10873C>T	p.(Gln3625*)	g.31164456G>A	1	-	[54]
76	c.10888C>T	p.(Arg3630*)	g.31164441G>A	1	-	[9]
76	c.10910C>A	p.(Ser3637*)	g.31164419G>T	4	-	[61]

**Table 3:** Patients with nonsense mutations in the same exons but with discordant phenotypes.

DMD EXON	FRAME	TOT		DMD		BMD		DMD/BMD		Not Defined		Other	
		Nonsense mutations	Patients	Nonsense mutations	Patients	Nonsense mutations	Patients	Nonsense mutations	Patients	Nonsense mutations	Patients	Nonsense mutations	Patients
1	-	4	35	1	1	3	34	0	0	0	0	0	0
2	out	4	5	0	0	3	3	0	0	1	2	0	0
3	in	5	16	2	12	2	3	1	1	0	0	0	0
5	in	7	18	3	4	1	3	3	11	0	0	0	0
21	out	18	44	12	37	1	2	1	1	3	3	1	1
25	in	18	58	7	15	6	14	4	27	1	2	0	0
26	in	21	48	13	37	1	1	1	1	5	8	1	1
27	in	13	19	9	14	2	2	0	0	1	1	1	2
28	in	12	24	5	10	2	4	3	8	0	0	2	2
29	in	12	57	4	8	4	34	1	10	3	5	0	0
31	in	7	12	0	0	4	9	2	2	1	1	0	0
33	in	14	32	11	27	1	1	1	1	0	0	1	3
35	in	15	55	8	48	3	3	2	2	2	2	0	0
37	in	10	39	8	16	2	23	0	0	0	0	0	0
38	in	9	34	2	4	3	13	4	17	0	0	0	0
39	in	16	65	8	49	2	3	1	5	3	3	2	5
40	in	16	34	9	21	1	1	3	6	2	2	1	4
41	in	20	76	10	58	1	1	4	11	4	4	1	2
49	in	5	10	1	1	1	5	1	1	2	3	0	0
56	out	8	11	6	9	1	1	1	1	0	0	0	0
72	in	2	10	0	0	1	7	1	3	0	0	0	0
73	in	2	2	0	0	1	1	0	0	1	1	0	0
74	in	10	18	4	5	3	4	2	8	1	1	0	0
75	out	8	14	3	7	2	2	1	3	1	1	1	1
76	out	6	13	2	6	3	6	1	1	0	0	0	0

For each exon, the fraction of blue color is proportional to the percentage of independent BMD cases with nonsense mutations, while the fraction of orange color is proportional to the percentage of DMD cases; exons without nonsense mutations are in gray. For each exon, the shape of box extremities represents the phase, where in-frame junctions are indicated by vertical lines.An arrow shape represents an exon starting (or ending) at the 2^nd^ or 3^rd^ nucleotide of a codon. Methionines in exons 6 and 8 are reported with a green circle.

### N-terminus

The distribution of nonsense mutations along the dystrophin molecule (Fig 2) is also quite surprising. Although it may be expected that the effect of a mutation at the beginning of the nascent polypeptide chain can be recovered from a re-initiation phenomenon [63], it is not clear how this can occur much further downstream. After the first start codon, the following methionines are two in exon 6 (position 124 and 128) [38] and three in exon 8 (230, 253 and 272) [64]. This could explain why nonsense mutations in exon 1, 2, 3 and 5 may be also associated with non-DMD phenotypes, but not why exon 4 mutations appear to be 100% DMD-linked.

### C-terminus

At the 3’ end, premature stop codons are understandably associated with milder phenotypes, because major part of the proteins has already been produced and therefore the truncated products may be partially functional. This prediction is supported by nonsense mutations of exons 72–76, also considering that most 3’ DMD exons are alternatively spliced [58, 65, 66]. No nonsense mutation in exons 77, 78 and 79 has been so far described in DMD/BMD patients. Recently, the last gnomAD v.3 reports a nonsense variant at position p.Arg3681* in exon 78, found in six African individuals (5 females and 1 male), and reported as variant of uncertain significance [67, 68].

This could suggest that nonsense mutations at the last 3’ end of the gene are not deleterious for the dystrophin function.

### Internal rod (in-frame exons)

A nonsense mutation in the middle of an open reading frame (ORF) generally undergoes nonsense mediated (mRNA) decay, a translation-coupled mechanism that eliminates mRNAs containing premature translation-termination codons [69]. Thus, even if it is possible a therapeutic induction of translation readthrough, the mRNA is degraded and therefore the expected phenotype should be severe. It is overly complex to measure the percentage of reduction of transcripts from muscle tissue in relation to the position of each nonsense mutation, but it seems clear that in many cases the phenomenon could be not stringent. Indeed, alternatively spliced isoforms could be actively selected by this mechanism, enriching the mRNA fraction with an ORF compared to those with stop codons.

On the other hand, if a portion of mRNA skips the exon with a mutation, a smaller protein could still be produced on the condition that the skipped exon is in-frame. Previous works hypothesized that mutations in in-frame exons might cause milder phenotypes via spontaneous exon skipping of the mutated exon, which may weaken the mutation consequence [14, 37]. This favorable precondition is the rule for most central dystrophin exons: all of them between 23 and 42 are in-frame. Apart from exon 29 that is alternatively spliced in normal muscle, all these other exons appear to be required [70]. Interestingly, consecutive exons may have divergent phenotypic associations. The skipping could restore the transcript and several reports have demonstrated that specific nonsense mutation can convert exonic splicing enhancer sequences (ESE) to silencer elements (ESS) [37, 71–73]. However, the situation is very strange for some exons such as 25, 31, 37 and 38 where many different nonsense mutations all lead to a mild phenotype (Table 2) [8, 45, 46, 56, 58, 61]. What is the explanation? Are these four exons easily skippable and thus are lost wherever they are mutated?

In addition, there is also the possibility of a multiple exon skipping. Nonsense mutations in the exon 27 cause the skipping of the exons 27–29 [53, 54]. Finally, it has been described that nonsense mutations in the exon 28 and 29 induce the skipping of single involved exon or the skipping of double exons (exons 28–29); moreover, mutations in the exon 39 cause 38–39 exons skipping [28, 42, 51, 56].

### Internal rod (out-of-frame exons)

**Fig 3:**
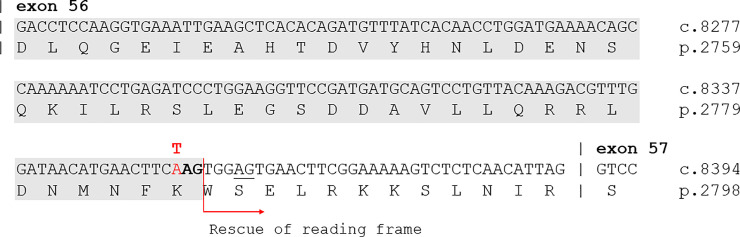
Prediction of a cryptic acceptor splice site in *DMD* exon56.

The explanation remains obscure for a few cases in out-of-frame exons. To provide an hypothesis, we checked two BMD-associated nonsense mutations in exons 21 and 56. The splice-site predictor software HSF [34] indicates that c.8353A>T, p.Lys2785*, in the exon 56, could cause the creation of two new splice acceptor sites. Only one allows to maintain the protein frame, thus explaining the BMD phenotype (Fig 3). By contrast, investigating the consequence of the variation c.2704C>T, p.Gln902*, in the exon 21, no splicing alteration was predicted. It is possible that self-correcting exon skipping may involve more than one exon and in this case prediction of phenotype effect based on small mutations location is not possible [74]. Therefore, to explain the reported association with BMD phenotype, it could be speculated about a potential coupled skipping of the exons 21–22, which could restore the protein frame (Fig 2).

By analyzing the c.8353A>T (p.Lys2785*) variant using the splice-site predictor software HSF, the nucleotide change is predicted to activate a cryptic acceptor splice site able to partially rescue reading-frame of exon 56, retaining the last 12 amino acids. A second weaker cryptic acceptor splice site is underscored, no rescuing reading-frame.

## Conclusions

By positioning all reported nonsense mutations along the dystrophin transcript, we observed a skewed concentration of BMD within selected exons. Previous data from large cohort of patients, [37, 61] and the present survey show that a milder than expected phenotype can be produced by the spontaneous elimination of a nonsense mutation from dystrophin mRNA in some central exons. The reported exceptions further confirm that natural mechanisms for rescue do exist. The observation suggests that exon skipping in the specific exons identified in this work could be a biologically more favored therapeutic approach than recovering deletions. Antisense oligonucleotides (AON) or new molecules, designed to induce the jump of specific exons are desirable. While on another fifty exons on the effects of the readthrough strategies can be more easily monitored.

Our graphical output may be of practical use both in genetic counselling and in recruitment of patients for translational readthrough of premature termination codons. From a more general point of view, our data confirm that multiple mechanisms can partially rescue nonsense mutations that should be not necessarily classified as null variants. This should be considered for the interpretation of NGS results.

## Supporting information

S1 Table Cohort of internal patients with nonsense mutations in *DMD* gene. (DOCX)

S2 Table 849 unique nonsense mutations from LOVD, HGMD, ClinVar and cohort of internal patients. (DOCX)
