# Supplementary material for: The position of nonsense mutations can predict the phenotype severity: A survey on the DMD gene
Source: PLoS One. 2020 Aug 19;15(8):e0237803. doi: 10.1371/journal.pone.0237803 (PMC7437896; doi:10.1371/journal.pone.0237803)
Supplement: S1 Table — (DOCX) [file pone.0237803.s001.docx]

**Table S1. Cohort of internal patients with nonsense mutations in *DMD* gene.**

| **Exon** | **DNAchange** | **Protein change** | **Disease** | **Reference** |
| --- | --- | --- | --- | --- |
| 5 | c.313A>T | p.(Lys105*) | BMD | [1] |
| 6 | c.433C>T | p.(Arg145*) | DMD | [1] |
| 6 | c.433C>T | p.(Arg145*) | DMD | [2] |
| 6 | c.433C>T | p.(Arg145*) | DMD | [2] |
| 6 | c.433C>T | p.(Arg145*) | DMD | [2] |
| 7 | c.583C>T | p.(Arg195*) | DMD | [2] |
| 8 | c.724C>T | p.(Gln242*) | DMD | [2] |
| 8 | c.701C>A | p.(Ser234*) | DMD | [2] |
| 8 | c.701C>A | p.(Ser234*) | DMD | [2] |
| 8 | c.724C>T | p.(Gln242*) | DMD | [1, 3] |
| 10 | c.1093C>T | p.(Gln365*) | DMD | [2] |
| 10 | c.1062G>A | p.(Trp354*) | DMD | [1] |
| 10 | c.1062G>A | p.(Trp354*) | DMD | [2] |
| 10 | c.1062G>A | p.(Trp354*) | DMD | [2] |
| 10 | c.1132C>T | p.(Gln378*) | DMD | Unpublished |
| 11 | c.1292G>A | p.(Trp431*) | DMD | [2] |
| 14 | c.1652G>A | p.(Trp551*) | DMD | [2] |
| 16 | c.1865C>G | p.(Ser622*) | DMD | [1] |
| 16 | c.1873C>T | p.(Gln625*) | DMD | Unpublished |
| 17 | c.2125C>T | p.(Gln709*) | DMD | [2] |
| 17 | c.2077T>C | p.(Gln693*) | DMD | [2] |
| 18 | c.2201G>A | p.(Trp734*) | DMD | Unpublished |
| 20 | c.2414C>G | p.(Ser805*) | DMD | [2] |
| 23 | c.3151C>T | p.(Arg1051*) | DMD | [2] |
| 23 | c.3151C>T | p.(Arg1051*) | DMD | Unpublished |
| 24 | C.3242C>A | p.(Ser1081*) | DMD | [2] |
| 24 | c.3259C>T | p.(Gln1087*) | DMD | [1] |
| 26 | c.3580C>T | p.(Gln1194*) | DMD | Unpublished |
| 28 | c.3843G>A | p.(Trp1281*) | DMD | [2] |
| 29 | c.3940C>T | p.(Arg1314*) | BMD | [2] |
| 29 | c.3940C>T | p.(Arg1314*) | DMD | [2] |
| 29 | c.3940C>T | p.(Arg1314*) | DMD | [2] |
| 29 | c.4027G>T | p.(Glu1343*) | DMD | [2] |
| 29 | c.3940C>T | p.(Arg1314*) | DMD | [2] |
| 29 | c.3940C>T | p.(Arg1314*) | DMD | [2] |
| 29 | c.3940C>T | p.(Arg1340*) | BMD | [2] |
| 29 | c.3940C>T | p.(Arg1314*) | BMD | [2] |
| 30 | c.4117C>T | p.(Gln1373*) | DMD | [2] |
| 34 | c.4729C>T | p.(Arg1577*) | DMD | [2] |
| 35 | c.4996C>T | p.(Arg1666*) | DMD | [2] |
| 35 | c.4979G>A | p.(Trp1660*) | BMD | [2] |
| 37 | c.5209C>T | p.(Gln1737*) | DMD | [2] |
| 39 | c.5530C>T | p.(Arg1844*) | DMD | [2] |
| 41 | c.5773G>T | p.(Glu1925*) | BMD | [2] |
| 41 | c.5773G>T | p.(Glu1925*) | BMD | Unpublished |
| 41 | c.5899C>T | p.(Arg1967*) | BMD | [1] |
| 41 | c.5899C>T | p.(Arg1967*) | DMD | [2] |
| 41 | c.5899C>T | p.(Arg1967*) | DMD | [2] |
| 41 | c.5899C>T | p.(Arg1967*) | DMD | [2] |
| 42 | c.6023C>A | p.(Ser2008*) | DMD | [2] |
| 42 | c.6103G>T | p.(Glu2035*) | DMD | Unpublished |
| 48 | c.7006C>T | p.(Gln2336*) | DMD | [2] |
| 59 | c.8713C>T | p.(Arg2905*) | DMD | [2] |
| 59 | c.8713C>T | p.(Arg2905*) | DMD | Unpublished |
| 60 | c.8944C>T | p.(Arg2982*) | DMD | [2] |
| 65 | c.9461T>A | p.(Leu3154*) | DMD | [2] |
| 65 | c.9474T>G | p.(Tyr3158*) | DMD | [2] |
| 65 | c.9558T>G | p.(Tyr3186*) | DMD | [2] |
| 70 | c.10108C>T | p.(Arg3370*) | DMD | [2] |
| 70 | c.10141C>T | p.(Arg3381*) | DMD | [2] |
| 76 | c.10801C>T | p.(Gln3601*) | BMD | Unpublished |

**References**

1. Nigro V, Nigro G, Esposito MG, Comi LI, Molinari AM, Puca GA, et al. Novel small mutations along the DMD/BMD gene associated with different phenotypes. Hum Mol Genet. 1994;3(10):1907-8. Epub 1994/10/01. doi: 10.1093/hmg/3.10.1907. PubMed PMID: 7849724.

2. Torella A, Trimarco A, Blanco Fdel V, Cuomo A, Aurino S, Piluso G, et al. One hundred twenty-one dystrophin point mutations detected from stored DNA samples by combinatorial denaturing high-performance liquid chromatography. J Mol Diagn. 2010;12(1):65-73. Epub 2009/12/05. doi: 10.2353/jmoldx.2010.090074. PubMed PMID: 19959795; PubMed Central PMCID: PMCPMC2797720.

3. Nigro V, Politano L, Nigro G, Romano SC, Molinari AM, Puca GA. Detection of a nonsense mutation in the dystrophin gene by multiple SSCP. Hum Mol Genet. 1992;1(7):517-20. Epub 1992/10/01. doi: 10.1093/hmg/1.7.517. PubMed PMID: 1307253.
