# Supplementary material for: The position of nonsense mutations can predict the phenotype severity: A survey on the DMD gene
Source: PLoS One. 2020 Aug 19;15(8):e0237803. doi: 10.1371/journal.pone.0237803 (PMC7437896; doi:10.1371/journal.pone.0237803)
Supplement: S2 Table — (DOCX) [file pone.0237803.s002.docx]

**Table S2. 849 unique nonsense mutations from LOVD, HGMD, ClinVar and cohort of internal patients.**

| **EXON** | **CDS** | **PROTEIN** | **hg19** | **Reference** | **Disease** | **Patients**  **(from LOVD)** |
| --- | --- | --- | --- | --- | --- | --- |
| 1 | c.8G>A | p.(Trp3*) | g.33229422C>T | [1] | BMD | 1 |
| 1 | c.9G>A | p.[Trp3*, Leu2_Met124del, Leu2_Met128del] | g.33229421C>T | [2] | BMD | 27 |
| 1 | c.11G>A | p.(Trp4*) | g.33229419C>T | [3, 4] | BMD | 6 |
| 1 | c.12G>A | p.(Trp4*) | g.33229418C>T | [3] | DMD | 1 |
| 2 | c.49C>T | p.(Gln17*) | g.33038300G>A | [5] | BMD | 1 |
| 2 | c.67A>T | p.(Lys23*) | g.33038282T>A | [1] | BMD | 1 |
| 2 | c.72G>A | p.(Trp24*) | g.33038277C>T | [6] | BMD | 1 |
| 2 | c.82C>T | p.(Gln28*) | g.33038267G>A | [2] | ND | 2 |
| 3 | c.100A>T | p.(Lys34*) | g.32867931T>A | [7] | DMD/BMD | 1 |
| 3 | c.103C>T | p.(Gln35*) | g.32867928G>A | [8] | BMD | 2 |
| 3 | c.133C>T | p.(Gln45*) | g.32867898G>A | [4, 9] | DMD | 9 |
| 3 | c.163G>T | p.[Glu55*, Phe32Metfs*13] | g.32867868C>A | [10] | BMD | 1 |
| 3 | c.178C>T | p.(Gln60*) | g.32867853G>A | [11] | DMD | 3 |
| 4 | c.193G>T | p.(Glu65*) | g.32862971C>A | [12] | DMD | 1 |
| 4 | c.196A>T | p.(Lys66*) | g.32862968T>A | [13] | DMD | 1 |
| 4 | c.199G>T | p.(Gly67*) | g.32862965C>A | [14] | DMD | 4 |
| 4 | c.253C>T | p.(Gln85*) | g.32862911G>A | [11] | DMD | 4 |
| 5 | c.272T>A | p.(Leu91*) | g.32841497A>T | [15] | DMD | 1 |
| 5 | c.283G>T | p.(Gly95*) | g.32841486C>A | [16] | DMD/BMD | 1 |
| 5 | c.313A>T | p.(Lys105*) | g.32841456T>A | [17] | DMD/BMD | 3 |
| 5 | c.336G>A | p.(Trp112*) | g.32841433C>T | [3] | BMD | 3 |
| 5 | c.353G>A | p.(Trp118*) | g.32841416C>T | [4] | DMD | 1 |
| 5 | c.354G>A | p.(Trp118*) | g.32841415C>T | [18] | DMD | 2 |
| 5 | c.355C>T | p.(Gln119*) | g.32841414G>A | [19] | DMD/BMD | 7 |
| 6 | c.361A>T | p.(Lys121*) | g.32834754T>A | [20] | DMD/BMD | 1 |
| 6 | c.394C>T | p.(Gln132*) | g.32834721G>A | [21] | DMD | 2 |
| 6 | c.397C>T | p.(Gln133*) | g.32834718G>A | [1] | DMD | 1 |
| 6 | c.409G>T | p.(Glu137*) | g.32834706C>A | [22] | DMD | 2 |
| 6 | c.428G>A | p.(Trp143*) | g.32834687C>T | [13] | DMD | 1 |
| 6 | c.429G>A | p.(Trp143*) | g.32834686C>T | [19] | DMD | 1 |
| 6 | c.433C>T | p.(Arg145*) | g.32834682G>A | [19, 23] | DMD | 38 |
| 6 | c.436C>T | p.(Gln146*) | g.32834679G>A | [24] | DMD | 5 |
| 6 | c.440C>A | p.(Ser147*) | g.32834675G>T | [25] | DMD | 1 |
| 6 | c.440C>G | p.(Ser147*) | g.32834675G>C | [19, 26] | DMD | 4 |
| 6 | c.453T>A | p.(Tyr151*) | g.32834662A>T | [27] | DMD | 1 |
| 6 | c.457C>T | p.(Gln153*) | g.32834658G>A | [19] | DMD | 2 |
| 6 | c.488G>A | p.(Trp163*) | g.32834627C>T | [28] | DMD | 3 |
| 6 | c.489G>A | p.(Trp163*) | g.32834626C>T | [1] | DMD/BMD | 1 |
| 7 | c.549G>A | p.(Trp183*) | g.32827710C>T | [19] | DMD/BMD | 1 |
| 7 | c.564C>A | p.(Cys188*) | g.32827695G>T | [29] | DMD | 1 |
| 7 | c.565C>T | p.(Gln189*) | g.32827694G>A | [30] | DMD | 4 |
| 7 | c.568C>T | p.(Gln190*) | g.32827691G>A | [19] | DMD | 10 |
| 7 | c.572C>G | p.(Ser191*) | g.32827687G>C | [28] | DMD | 4 |
| 7 | c.580C>T | p.(Gln194*) | g.32827679G>A | [19, 21] | DMD | 5 |
| 7 | c.583C>T | p.(Arg195*) | g.32827676G>A | [10] | DMD | 37 |
| 7 | c.589G>T | p.(Glu197*) | g.32827670C>A | [31] | ND | 1 |
| 7 | c.615T>A | p.(Tyr205*) | g.32827644A>T | [25] | DMD | 3 |
| 7 | c.616C>T | p.(Gln206*) | g.32827643G>A | [32] | DMD | 1 |
| 7 | c.620T>G | p.(Leu207*) | g.32827639A>C | [20] | DMD | 3 |
| 7 | c.646G>T | p.(Glu216*) | g.32827613C>A | [1] | DMD | 1 |
| 8 | c.673A>T | p.(Lys225*) | g.32717387T>A | [12] | DMD | 1 |
| 8 | c.686T>G | p.(Leu229*) | g.32717374A>C | [16] | DMD | 1 |
| 8 | c.686T>A | p.(Leu229*) | g.32717374A>T | [19] | OTHER | 2 |
| 8 | c.693C>A | p.(Tyr231*) | g.32717367G>T | [4] | DMD | 1 |
| 8 | c.701C>A | p.(Ser234*) | g.32717359G>T | [33] | DMD | 6 |
| 8 | c.701C>G | p.(Ser234*) | g.32717359G>C | [13] | DMD | 1 |
| 8 | c.709C>T | p.(Gln237*) | g.32717351G>A | [34] | DMD | 4 |
| 8 | c.721C>T | p.(Gln241*) | g.32717339G>A | [10] | DMD | 2 |
| 8 | c.724C>T | p.(Gln242*) | g.32717336G>A | [35] | DMD | 9 |
| 8 | c.745C>T | p.(Gln249*) | g.32717315G>A | [36] | DMD | 3 |
| 8 | c.748G>T | p.(Glu250*) | g.32717312C>A | [11] | DMD | 2 |
| 8 | c.754G>T | p.(Glu252*) | g.32717306C>A | [34] | DMD | 3 |
| 8 | c.775A>T | p.(Lys259*) | g.32717285T>A | [20] | DMD | 1 |
| 8 | c.799C>T | p.(Gln267*) | g.32717261G>A | [19] | DMD | 4 |
| 8 | c.811C>T | p.(Gln271*) | g.32717249G>A | [20] | DMD | 3 |
| 8 | c.826C>T | p.(Gln276*) | g.32717234G>A | [3] | DMD | 2 |
| 8 | c.829C>T | p.(Gln277*) | g.32717231G>A | [37][3] | DMD | 9 |
| 9 | c.853G>T | p.(Gly285*) | g.32716094C>A | [13, 38] | DMD | 4 |
| 9 | c.858T>G | p.(Tyr286*) | g.32716089A>C | [1] | DMD | 1 |
| 9 | c.883C>T | p.[Arg295*,Ile278_Gln320del] | g.32716064G>A | [38] | DMD/BMD | 9 |
| 9 | c.897T>G | p.[Tyr299*; Ile278_Gln320del] | g.32716050A>C | [1] | DMD | 1 |
| 9 | c.903C>A | p.(Tyr301*) | g.32716044G>T | [39] | DMD | 1 |
| 9 | c.903C>G | p.(Tyr301*) | g.32716044G>C | [19] | DMD | 2 |
| 9 | c.907C>T | p.(Gln303*) | g.32716040G>A | [28] | ND | 2 |
| 9 | c.956C>G | p.(Ser319*) | g.32715991G>C | [39] | DMD | 1 |
| 10 | c.965T>A | p.(Leu322*) | g.32663265A>T | [25] | DMD | 1 |
| 10 | c.986C>G | p.(Ser329*) | g.32663244G>C | [1] | ND | 1 |
| 10 | c.998C>A | p.(Ser333*) | g.32663232G>T | [25] | DMD | 5 |
| 10 | c.1006G>T | p.(Glu336*) | g.32663224C>A | [40] | DMD | 2 |
| 10 | c.1012G>T | p.(Glu338*) | g.32663218C>A | [19] | DMD | 1 |
| 10 | c.1033C>T | p.(Gln345*) | g.32663197G>A | [20] | DMD | 3 |
| 10 | c.1045G>T | p.(Glu349*) | g.32663185C>A | [1] | DMD | 1 |
| 10 | c.1048G>T | p.(Glu350*) | g.32663182C>A | [1] | DMD/BMD | 2 |
| 10 | c.1055T>G | p.(Leu352*) | g.32663175A>C | [41] | DMD | 2 |
| 10 | c.1061G>A | p.(Trp354*) | g.32663169C>T | [13, 19] | DMD/BMD | 4 |
| 10 | c.1062G>A | p.(Trp354*) | g.32663168C>T | [10] | DMD | 5 |
| 10 | c.1075G>T | p.(Glu359*) | g.32663155C>A | [16] | ND | 1 |
| 10 | c.1087C>T | p.(Gln363*) | g.32663143G>A | [18] | DMD | 1 |
| 10 | c.1093C>T | p.(Gln365*) | g.32663137G>A | [22] | DMD | 4 |
| 10 | c.1099G>T | p.(Glu367*) | g.32663131C>A | [21] | DMD | 1 |
| 10 | c.1117G>T | p.(Glu373*) | g.32663113C>A | [19] | DMD | 3 |
| 10 | c.1132C>T | p.(Gln378*) | g.32663098G>A | [28] | DMD | 2 |
| 11 | c.1177C>T | p.(Gln393*) | g.32662403G>A | [21] | DMD | 2 |
| 11 | c.1201C>T | p.(Gln401*) | g.32662379G>A | [7] | DMD/BMD | 2 |
| 11 | c.1207G>T | p.(Gly403*) | g.32662373C>A | [37] | ND | 1 |
| 11 | c.1238C>A | p.(Ser413*) | g.32662342G>T | [1] | DMD | 1 |
| 11 | c.1238C>G | p.(Ser413*) | g.32662342G>C | [42] | DMD | 1 |
| 11 | c.1249G>T | p.(Glu417*) | g.32662331C>A | [23] | DMD | 1 |
| 11 | c.1255G>T | p.(Glu419*) | g.32662325C>A | [32] | DMD | 2 |
| 11 | c.1261C>T | p.(Gln421*) | g.32662319G>A | [26] | DMD | 4 |
| 11 | c.1264G>T | p.(Glu422*) | g.32662316C>A | [1] | DMD | 1 |
| 11 | c.1267C>T | p.(Gln423*) | g.32662313G>A | [19] | DMD | 6 |
| 11 | c.1286C>A | p.(Ser429*) | g.32662294G>T | [19] | DMD/BMD | 2 |
| 11 | c.1286C>G | p.(Ser429*) | g.32662294G>C | [1] | DMD/BMD | 1 |
| 11 | c.1288A>T | p.(Arg430*) | g.32662292T>A | [16] | ND | 1 |
| 11 | c.1292G>A | p.(Trp431*) | g.32662288C>T | [25] | DMD | 3 |
| 11 | c.1293G>A | p.(Trp431*) | g.32662287C>T | [1] | DMD | 2 |
| 11 | c.1324C>T | p.(Gln442*) | g.32662256G>A | [10] | DMD | 1 |
| 12 | c.1334T>G | p.(Leu445*) | g.32632568A>C | [1] | DMD | 1 |
| 12 | c.1339A>T | p.(Arg447*) | g.32632563T>A | [13] | DMD | 2 |
| 12 | c.1357C>T | p.(Gln453*) | g.32632545G>A | [13, 37] | DMD | 4 |
| 12 | c.1363C>T | p.(Gln455*) | g.32632539G>A | [3, 21] | DMD | 3 |
| 12 | c.1366A>T | p.(Lys456*) | g.32632536T>A | [1] | DMD | 1 |
| 12 | c.1372A>T | p.(Lys458*) | g.32632530T>A | [13] | DMD | 1 |
| 12 | c.1388G>A | p.(Trp463*) | g.32632514C>T | [13, 19] | DMD | 4 |
| 12 | c.1389G>A | p.(Trp463*) | g.32632513C>T | [1] | DMD | 1 |
| 12 | c.1417A>T | p.(Lys473*) | g.32632485T>A | [16] | DMD/BMD | 1 |
| 12 | c.1426G>T | p.(Glu476*) | g.32632476C>A | [23] | DMD | 2 |
| 12 | c.1429G>T | p.(Glu477*) | g.32632473C>A | [19] | DMD/BMD | 3 |
| 12 | c.1438G>T | p.(Gly480*) | g.32632464C>A | [4] | DMD/BMD | 2 |
| 12 | c.1465C>T | p.(Gln489*) | g.32632437G>A | [10] | DMD/BMD | 4 |
| 12 | c.1471C>T | p.(Gln491*) | g.32632431G>A | [1] | ND | 1 |
| 12 | c.1474C>T | p.(Gln492*) | g.32632428G>A | [18, 26] | DMD | 4 |
| 13 | c.1489C>T | p.(Gln497*) | g.32613987G>A | [43] | DMD | 4 |
| 13 | c.1504C>T | p.(Gln502*) | g.32613972G>A | [13] | DMD | 1 |
| 13 | c.1507G>T | p.(Glu503*) | g.32613969C>A | [1] | OTHER | 1 |
| 13 | c.1510C>T | p.(Gln504*) | g.32613966G>A | [4, 44] | DMD | 4 |
| 13 | c.1555G>T | p.(Glu519*) | g.32613921C>A | [45] | DMD | 1 |
| 13 | c.1594C>T | p.(Gln532*) | g.32613882G>A | [30] | DMD | 6 |
| 14 | c.1609G>T | p.(Gly537*) | g.32591957C>A | [28] | ND | 1 |
| 14 | c.1615C>T | p.(Arg539*) | g.32591951G>A | [20, 40] | DMD | 21 |
| 14 | c.1619G>A | p.(Trp540*) | g.32591947C>T | [46] | DMD | 3 |
| 14 | c.1620G>A | p.(Trp540*) | g.32591946C>T | [1] | DMD | 1 |
| 14 | c.1633A>T | p.(Arg545*) | g.32591933T>A | [12] | DMD | 1 |
| 14 | c.1637G>A | p.(Trp546*) | g.32591929C>T | [37] | DMD | 3 |
| 14 | c.1638G>A | p.(Trp546*) | g.32591928C>T | [3, 23, 43] | DMD | 4 |
| 14 | c.1642G>T | p.(Glu548*) | g.32591924C>A | [1] | DMD | 1 |
| 14 | c.1652G>A | p.(Trp551*) | g.32591914C>T | [13] | DMD | 4 |
| 14 | c.1653G>A | p.(Trp551*) | g.32591913C>T | [1] | DMD | 1 |
| 14 | c.1663C>T | p.(Gln555*) | g.32591903G>A | [46] | DMD | 15 |
| 14 | c.1682G>A | p.(Trp561*) | g.32591884C>T | [30] | DMD | 1 |
| 14 | c.1683G>A | p.(Trp561*) | g.32591883C>T | [25] | DMD | 3 |
| 14 | c.1684C>T | p.(Gln562*) | g.32591882G>A | [18] | DMD | 3 |
| 14 | c.1699G>T | p.(Glu567*) | g.32591867C>A | [28] | ND | 1 |
| 14 | c.1702C>T | p.(Gln568*) | g.32591864G>A | [34] | DMD | 9 |
| 15 | c.1707C>A | p.(Cys569*) | g.32591752G>T | [13] | DMD/BMD | 2 |
| 15 | c.1721G>A | p.(Trp574*) | g.32591738C>T | [47] | DMD | 2 |
| 15 | c.1727C>A | p.(Ser576*) | g.32591732G>T | [19] | DMD/BMD | 2 |
| 15 | c.1729G>T | p.(Glu577*) | g.32591730C>A | [7] | DMD/BMD | 2 |
| 15 | c.1732A>T | p.(Lys578*) | g.32591727T>A | [13] | DMD | 2 |
| 15 | c.1777C>T | p.(Gln593*) | g.32591682G>A | [3] | DMD | 2 |
| 15 | c.1783G>T | p.(Glu595*) | g.32591676C>A | [19] | DMD | 3 |
| 15 | c.1793C>G | p.(Ser598*) | g.32591666G>C | [18, 19] | DMD | 3 |
| 16 | c.1817T>G | p.(Leu606*) | g.32583994A>C | [1] | DMD | 2 |
| 16 | c.1831G>T | p.(Glu611*) | g.32583980C>A | [7] | DMD/BMD | 1 |
| 16 | c.1837A>T | p.(Lys613*) | g.32583974T>A | [21] | DMD | 1 |
| 16 | c.1843C>T | p.(Gln615*) | g.32583968G>A | [3, 23] | DMD | 3 |
| 16 | c.1865C>G | p.(Ser622*) | g.32583946G>C | [17] | DMD | 2 |
| 16 | c.1865C>A | p.(Ser622*) | g.32583946G>T | [16] | ND | 1 |
| 16 | c.1873C>T | p.(Gln625*) | g.32583938G>A | [48] | DMD | 1 |
| 16 | c.1886C>G | p.(Ser629*) | g.32583925G>C | [49] | DMD | 3 |
| 16 | c.1886C>A | p.(Ser629*) | g.32583925G>T | [16] | ND | 1 |
| 16 | c.1900A>T | p.(Lys634*) | g.32583911T>A | [1] | DMD/BMD | 1 |
| 16 | c.1904C>G | p.(Ser635*) | g.32583907G>C | [20] | DMD | 2 |
| 16 | c.1912C>T | p.(Gln638*) | g.32583899G>A | [7] | DMD/BMD | 1 |
| 16 | c.1928G>A | p.(Trp643*) | g.32583883C>T | [50] | DMD | 2 |
| 16 | c.1952G>A | p.(Trp651*) | g.32583859C>T | [11] | DMD | 2 |
| 16 | c.1961T>A | p.(Leu654*) | g.32583850A>T | [19] | DMD | 2 |
| 16 | c.1961T>G | p.(Leu654*) | g.32583850A>C | [1] | DMD/BMD | 2 |
| 16 | c.1966C>T | p.(Gln656*) | g.32583845G>A | [19] | DMD | 3 |
| 16 | c.1975G>T | p.(Glu659*) | g.32583836C>A | [1] | DMD | 1 |
| 16 | c.1990C>T | p.(Gln664*) | g.32583821G>A | [46] | DMD | 7 |
| 17 | c.1997C>A | p.(Ser666*) | g.32563447G>T | [3] | DMD | 1 |
| 17 | c.1999C>T | p.(Gln667*) | g.32563445G>A | [1] | DMD | 1 |
| 17 | c.2017C>T | p.(Gln673*) | g.32563427G>A | [13] | DMD | 3 |
| 17 | c.2032C>T | p.(Gln678*) | g.32563412G>A | [4, 13] | DMD | 5 |
| 17 | c.2047G>T | p.(Glu683*) | g.32563397C>A | [18] | DMD | 3 |
| 17 | c.2074G>T | p.(Glu692*) | g.32563370C>A | [21] | ND | 1 |
| 17 | c.2077C>T | p.(Gln693*) | g.32563367G>A | [19] | DMD | 5 |
| 17 | c.2089A>T | p.(Lys697*) | g.32563355T>A | [18] | DMD | 1 |
| 17 | c.2098C>T | p.(Gln700*) | g.32563346G>A | [51] | DMD | 1 |
| 17 | c.2101G>T | p.(Glu701*) | g.32563343C>A | [16] | OTHER | 1 |
| 17 | c.2104G>T | p.(Glu702*) | g.32563340C>A | [19] | OTHER | 3 |
| 17 | c.2125C>T | p.(Gln709*) | g.32563319G>A | [52] | DMD | 4 |
| 17 | c.2128A>T | p.(Lys710*) | g.32563316T>A | [30] | DMD | 1 |
| 17 | c.2137C>T | p.(Gln713*) | g.32563307G>A | [53] | DMD | 4 |
| 18 | c.2201G>A | p.(Trp734*) | g.32536216C>T | Internal patient | DMD | 1 |
| 18 | c.2202G>A | p.(Trp734*) | g.32536215C>T | [16] | ND | 1 |
| 18 | c.2213C>G | p.(Ser738*) | g.32536204G>C | [7] | DMD/BMD | 3 |
| 18 | c.2215G>T | p.(Glu739*) | g.32536202C>A | [1] | DMD | 1 |
| 18 | c.2227C>T | p.(Gln743*) | g.32536190G>A | [4] | DMD | 6 |
| 18 | c.2236G>T | p.(Glu746*) | g.32536181C>A | [18] | DMD | 3 |
| 18 | c.2257G>T | p.(Glu753*) | g.32536160C>A | [23] | DMD | 2 |
| 18 | c.2270C>G | p.(Ser757*) | g.32536147G>C | [13, 48, 50] | DMD | 4 |
| 18 | c.2276T>A | p.(Leu759*) | g.32536141A>T | [16] | DMD | 1 |
| 18 | c.2276T>G | p.(Leu759*) | g.32536141A>C | [10] | DMD | 1 |
| 18 | c.2281G>T | p.(Glu761*) | g.32536136C>A | [10] | DMD | 1 |
| 19 | c.2299G>T | p.(Glu767*) | g.32519953C>A | [30] | DMD | 2 |
| 19 | c.2302C>T | p.(Arg768*) | g.32519950G>A | [3] | DMD | 33 |
| 19 | c.2308A>T | p.(Lys770*) | g.32519944T>A | [11, 28] | DMD | 4 |
| 19 | c.2314G>T | p.[Glu772*, Ala765Argfs*15] | g.32519938C>A | [1] | DMD | 2 |
| 19 | c.2317A>T | p.(Lys773*) | g.32519935T>A | [19] | DMD | 1 |
| 19 | c.2332C>T | p.(Gln778*) | g.32519920G>A | [37] | DMD | 2 |
| 19 | c.2353C>T | p.(Gln785*) | g.32519899G>A | [37] | DMD | 4 |
| 19 | c.2365G>T | p.(Glu789*) | g.32519887C>A | [18] | DMD | 3 |
| 19 | c.2368C>T | p.(Gln790*) | g.32519884G>A | [34] | DMD | 3 |
| 19 | c.2380G>T | p.(Glu794*) | g.32519872C>A | [52] | DMD | 1 |
| 20 | c.2392G>T | p.(Glu798*) | g.32509624C>A | [16] | ND | 1 |
| 20 | c.2404A>T | p.(Lys802*) | g.32509612T>A | [19] | DMD | 1 |
| 20 | c.2407C>T | p.(Gln803*) | g.32509609G>A | [50] | DMD | 3 |
| 20 | c.2414C>G | p.(Ser805*) | g.32509602G>C | [52] | ND | 1 |
| 20 | c.2416G>T | p.(Glu806*) | g.32509600C>A | [19] | DMD | 1 |
| 20 | c.2419C>T | p.(Gln807*) | g.32509597G>A | [18] | DMD/BMD | 5 |
| 20 | c.2435G>A | p.(Trp812*) | g.32509581C>T | [13, 19] | DMD | 4 |
| 20 | c.2436G>A | p.(Trp812*) | g.32509580C>T | [1] | DMD/BMD | 2 |
| 20 | c.2440G>T | p.(Glu814*) | g.32509576C>A | [19] | OTHER | 2 |
| 20 | c.2448C>A | p.(Cys816*) | g.32509568G>T | [13] | DMD | 2 |
| 20 | c.2449C>T | p.(Gln817*) | g.32509567G>A | [18] | DMD | 2 |
| 20 | c.2474G>A | p.(Trp825*) | g.32509542C>T | [54] | DMD | 1 |
| 20 | c.2475G>A | p.(Trp825*) | g.32509541C>T | [55] | DMD | 1 |
| 20 | c.2479G>T | p.(Glu827*) | g.32509537C>A | [19] | DMD | 3 |
| 20 | c.2484T>A | p.(Tyr828*) | g.32509532A>T | [23] | DMD | 1 |
| 20 | c.2484T>G | p.(Tyr828*) | g.32509532A>C | [1] | ND | 1 |
| 20 | c.2485C>T | p.(Gln829*) | g.32509531G>A | [19] | DMD | 1 |
| 20 | c.2512C>T | p.(Gln838*) | g.32509504G>A | [55] | DMD | 1 |
| 20 | c.2518C>T | p.(Gln840*) | g.32509498G>A | [4] | DMD | 2 |
| 20 | c.2521C>T | p.(Gln841*) | g.32509495G>A | [25] | DMD | 4 |
| 20 | c.2525T>A | p.(Leu842*) | g.32509491A>T | [1] | DMD | 1 |
| 20 | c.2527G>T | p.(Glu843*) | g.32509489C>A | [40] | DMD/BMD | 4 |
| 20 | c.2530C>T | p.(Gln844*) | g.32509486G>A | [56] | DMD | 2 |
| 20 | c.2548G>T | p.(Glu850*) | g.32509468C>A | [33] | OTHER | 1 |
| 20 | c.2555G>A | p.(Trp852*) | g.32509461C>T | [16] | ND | 1 |
| 20 | c.2558T>A | p.(Leu853*) | g.32509458A>T | [39] | DMD | 1 |
| 20 | c.2560A>T | p.(Lys854*) | g.32509456T>A | [1] | DMD | 1 |
| 20 | c.2566C>T | p.(Gln856*) | g.32509450G>A | [1] | DMD | 2 |
| 20 | c.2582C>G | p.(Ser861*) | g.32509434G>C | [19] | DMD | 1 |
| 20 | c.2584G>T | p.(Glu862*) | g.32509432C>A | [13] | DMD | 1 |
| 20 | c.2599A>T | p.(Lys867*) | g.32509417T>A | [1] | DMD | 1 |
| 20 | c.2605C>T | p.(Gln869*) | g.32509411G>A | [15] | DMD | 5 |
| 20 | c.2609T>G | p.(Leu870*) | g.32509407A>C | [20] | DMD/BMD | 1 |
| 20 | c.2611A>T | p.(Lys871*) | g.32509405T>A | [19] | DMD | 2 |
| 21 | c.2626G>T | p.(Glu876*) | g.32503213C>A | [1] | ND | 1 |
| 21 | c.2642C>G | p.(Ser881*) | g.32503197G>C | [1] | ND | 1 |
| 21 | c.2650C>T | p.(Gln884*) | g.32503189G>A | [25] | DMD | 3 |
| 21 | c.2656C>T | p.(Gln886*) | g.32503183G>A | [33] | OTHER | 1 |
| 21 | c.2665C>T | p.(Arg889*) | g.32503174G>A | [28, 37, 53] | DMD | 5 |
| 21 | c.2669T>G | p.(Leu890*) | g.32503170A>C | [15] | DMD | 2 |
| 21 | c.2669T>A | p.(Leu890*) | g.32503170A>T | [16] | DMD/BMD | 1 |
| 21 | c.2671A>T | p.(Lys891*) | g.32503168T>A | [12] | DMD | 1 |
| 21 | c.2677C>T | p.(Gln893*) | g.32503162G>A | [18] | DMD | 2 |
| 21 | c.2701G>T | p.(Gly901*) | g.32503138C>A | [25] | DMD | 1 |
| 21 | c.2704C>T | p.(Gln902*) | g.32503135G>A | [13] | BMD | 2 |
| 21 | c.2707G>T | p.(Gly903*) | g.32503132C>A | [28] | ND | 1 |
| 21 | c.2755A>T | p.(Lys919*) | g.32503084T>A | [57] | DMD | 2 |
| 21 | c.2758C>T | p.(Gln920*) | g.32503081G>A | [19] | DMD | 7 |
| 21 | c.2776C>T | p.(Gln926*) | g.32503063G>A | [45] | DMD | 3 |
| 21 | c.2788A>T | p.(Lys930*) | g.32503051T>A | [34] | DMD | 3 |
| 21 | c.2791G>T | p.(Glu931*) | g.32503048C>A | [58] | DMD | 2 |
| 21 | c.2797C>T | p.(Gln933*) | g.32503042G>A | [10] | DMD | 6 |
| 22 | c.2816T>A | p.(Leu939*) | g.32490414A>T | [53] | DMD | 3 |
| 22 | c.2832T>A | p.(Tyr944*) | g.32490398A>T | [3] | DMD | 1 |
| 22 | c.2832T>G | p.(Tyr944*) | g.32490398A>C | [59] | ND | 1 |
| 22 | c.2833C>T | p.(Gln945*) | g.32490397G>A | [14] | DMD | 4 |
| 22 | c.2836G>T | p.(Glu946*) | g.32490394C>A | [23] | DMD | 2 |
| 22 | c.2861G>A | p.(Trp954*) | g.32490369C>T | [16] | DMD | 1 |
| 22 | c.2862G>A | p.(Trp954*) | g.32490368C>T | [19] | DMD | 1 |
| 22 | c.2866C>T | p.(Gln956*) | g.32490364G>A | [49] | DMD/BMD | 5 |
| 22 | c.2869C>T | p.(Gln957*) | g.32490361G>A | [3, 28] | DMD | 5 |
| 22 | c.2873C>G | p.(Ser958*) | g.32490357G>C | [28] | ND | 1 |
| 22 | c.2916T>A | p.(Tyr972*) | g.32490314A>T | [1] | DMD | 1 |
| 22 | c.2926G>T | p.(Glu976*) | g.32490304C>A | [60] | ND | 1 |
| 22 | c.2929C>T | p.(Gln977*) | g.32490301G>A | [47] | DMD | 3 |
| 22 | c.2941G>T | p.(Glu981*) | g.32490289C>A | [1] | DMD | 2 |
| 22 | c.2947C>T | p.(Gln983*) | g.32490283G>A | [54] | DMD | 1 |
| 23 | c.2954T>A | p.(Leu985*) | g.32486823A>T | [34] | DMD | 2 |
| 23 | c.2956C>T | p.(Gln986*) | g.32486821G>A | [54] | DMD | 3 |
| 23 | c.2968C>T | p.(Gln990*) | g.32486809G>A | [30] | DMD | 5 |
| 23 | c.2971G>T | p.(Glu991*) | g.32486806C>A | [21, 42] | OTHER | 3 |
| 23 | c.2974C>T | p.(Gln992*) | g.32486803G>A | [28] | DMD | 3 |
| 23 | c.2977C>T | p.(Gln993*) | g.32486800G>A | [1] | DMD/BMD | 1 |
| 23 | c.2991C>G | p.(Tyr997*) | g.32486786G>C | [19] | DMD | 7 |
| 23 | c.2994T>A | p.(Tyr998*) | g.32486783A>T | [1] | DMD/BMD | 1 |
| 23 | c.3013G>T | p.(Glu1005*) | g.32486764C>A | [13] | DMD | 1 |
| 23 | c.3022A>T | p.(Lys1008*) | g.32486755T>A | [1] | DMD/BMD | 1 |
| 23 | c.3037G>T | p.(Glu1013*) | g.32486740C>A | [61] | DMD | 1 |
| 23 | c.3049A>T | p.(Lys1017*) | g.32486728T>A | [1] | DMD | 1 |
| 23 | c.3054T>G | p.(Tyr1018*) | g.32486723A>C | [19] | DMD/BMD | 1 |
| 23 | c.3055C>T | p.(Gln1019*) | g.32486722G>A | [62] | DMD | 2 |
| 23 | c.3059C>G | p.(Ser1020*) | g.32486718G>C | [1] | DMD/BMD | 2 |
| 23 | c.3061G>T | p.(Glu1021*) | g.32486716C>A | [39] | DMD | 1 |
| 23 | c.3067G>T | p.(Glu1023*) | g.32486710C>A | [1] | DMD | 2 |
| 23 | c.3076G>T | p.(Glu1026*) | g.32486701C>A | [63] | DMD/BMD | 3 |
| 23 | c.3079G>T | p.(Gly1027*) | g.32486698C>A | [13] | DMD | 1 |
| 23 | c.3086G>A | p.(Trp1029*) | g.32486691C>T | [28] | DMD | 2 |
| 23 | c.3087G>A | p.(Trp1029*) | g.32486690C>T | [3, 19] | DMD | 5 |
| 23 | c.3103C>T | p.(Gln1035*) | g.32486674G>A | [44] | DMD | 2 |
| 23 | c.3121C>T | p.(Gln1041*) | g.32486656G>A | [11, 19] | DMD | 7 |
| 23 | c.3124A>T | p.(Lys1042*) | g.32486653T>A | [1] | DMD/BMD | 1 |
| 23 | c.3136C>T | p.(Gln1046*) | g.32486641G>A | [19] | DMD | 4 |
| 23 | c.3151C>T | p.(Arg1051*) | g.32486626G>A | [3, 47] | DMD | 23 |
| 23 | c.3154A>T | p.(Lys1052*) | g.32486623T>A | [61] | DMD | 1 |
| 24 | c.3172C>T | p.(Gln1058*) | g.32482807G>A | [44] | DMD/BMD | 3 |
| 24 | c.3184A>T | p.(Lys1062*) | g.32482795T>A | [1] | DMD | 1 |
| 24 | c.3188G>A | p.(Trp1063*) | g.32482791C>T | [11] | DMD | 3 |
| 24 | c.3196G>T | p.(Glu1066*) | g.32482783C>A | [12] | DMD | 1 |
| 24 | c.3217G>T | p.(Glu1073*) | g.32482762C>A | [19] | DMD | 2 |
| 24 | c.3220G>T | p.(Glu1074*) | g.32482759C>A | [30] | DMD | 3 |
| 24 | c.3224G>A | p.(Trp1075*) | g.32482755C>T | [19] | DMD | 1 |
| 24 | c.3242C>A | p.(Ser1081*) | g.32482737G>T | [33] | ND | 2 |
| 24 | c.3244G>T | p.(Glu1082*) | g.32482735C>A | [45] | DMD | 3 |
| 24 | c.3253A>T | p.(Lys1085*) | g.32482726T>A | [23] | DMD | 1 |
| 24 | c.3256A>T | p.(Lys1086*) | g.32482723T>A | [64] | DMD | 3 |
| 24 | c.3259C>T | p.(Gln1087*) | g.32482720G>A | [17] | DMD | 8 |
| 24 | c.3268C>T | p.(Gln1090*) | g.32482711G>A | [28] | DMD | 2 |
| 24 | c.3274A>T | p.(Arg1092*) | g.32482705T>A | [12] | DMD | 1 |
| 25 | c.3281T>A | p.[Leu1094*; Leu1093_Gln1144del] | g.32481707A>T | [19] | DMD | 1 |
| 25 | c.3281T>G | p.(Leu1094*) | g.32481707A>C | [19] | DMD | 1 |
| 25 | c.3295C>T | p.[Gln1099*,Leu1093_Gln1144del] | g.32481693G>A | [38] | DMD/BMD | 7 |
| 25 | c.3304C>T | p.[Gln1102*,Leu1093_Gln1144del] | g.32481684G>A | [13, 38] | DMD/BMD | 6 |
| 25 | c.3328G>T | p.[Glu1110*, Leu1093_Gln1144del] | g.32481660C>A | [10] | BMD | 1 |
| 25 | c.3337C>T | p.[Gln1113*,Leu1093_Gln1144del] | g.32481651G>A | [13, 38] | DMD/BMD | 8 |
| 25 | c.3340A>T | p.(Lys1114*) | g.32481648T>A | [19] | BMD | 3 |
| 25 | c.3346A>T | p.(Lys1116*) | g.32481642T>A | [7] | ND | 2 |
| 25 | c.3352G>T | p.(Glu1118*) | g.32481636C>A | [27] | BMD | 2 |
| 25 | c.3358G>T | p.(Glu1120*) | g.32481630C>A | [56] | BMD | 1 |
| 25 | c.3376A>T | p.(Arg1126*) | g.32481612T>A | [65] | DMD | 1 |
| 25 | c.3397G>T | p.(Glu1133*) | g.32481591C>A | [66] | DMD | 4 |
| 25 | c.3409C>T | p.(Gln1137*) | g.32481579G>A | [22] | BMD | 3 |
| 25 | c.3413G>A | p.(Trp1138*) | g.32481575C>T | [34] | BMD | 4 |
| 25 | c.3414G>A | p.(Trp1138*) | g.32481574C>T | [23] | DMD | 3 |
| 25 | c.3426C>A | p.(Cys1142*) | g.32481562G>T | [3] | DMD | 2 |
| 25 | c.3427C>T | p.(Gln1143*) | g.32481561G>A | [39] | DMD | 3 |
| 25 | c.3430C>T | p.[Gln1144*,Leu1093_Gln1144del] | g.32481558G>A | [13, 38] | DMD/BMD | 6 |
| 26 | c.3445A>T | p.(Lys1149*) | g.32472937T>A | [20] | ND | 2 |
| 26 | c.3448G>T | p.(Glu1150*) | g.32472934C>A | [13] | DMD | 1 |
| 26 | c.3460G>T | p.(Gly1154*) | g.32472922C>A | [29] | DMD | 1 |
| 26 | c.3469G>T | p.(Glu1157*) | g.32472913C>A | [1] | DMD | 1 |
| 26 | c.3472A>T | p.(Lys1158*) | g.32472910T>A | [18] | DMD | 1 |
| 26 | c.3487C>T | p.(Gln1163*) | g.32472895G>A | [43] | DMD | 3 |
| 26 | c.3500C>G | p.(Ser1167*) | g.32472882G>C | [19] | DMD | 5 |
| 26 | c.3502G>T | p.(Glu1168*) | g.32472880C>A | [1] | ND | 1 |
| 26 | c.3511G>T | p.(Glu1171*) | g.32472871C>A | [23] | DMD | 3 |
| 26 | c.3515G>A | p.[Trp1172*, Val1145_Lys1201del] | g.32472867C>T | [15] | BMD | 1 |
| 26 | c.3516G>A | p.(Trp1172*) | g.32472866C>T | [47] | OTHER | 1 |
| 26 | c.3523C>T | p.(Gln1175*) | g.32472859G>A | [34] | DMD | 1 |
| 26 | c.3532G>T | p.(Glu1178*) | g.32472850C>A | [16] | DMD/BMD | 1 |
| 26 | c.3535G>T | p.(Glu1179*) | g.32472847C>A | [1] | ND | 3 |
| 26 | c.3544G>T | p.(Glu1182*) | g.32472838C>A | [19] | DMD | 1 |
| 26 | c.3556G>T | p.(Glu1186*) | g.32472826C>A | [27] | ND | 1 |
| 26 | c.3562A>T | p.(Lys1188*) | g.32472820T>A | [67] | DMD | 2 |
| 26 | c.3578T>A | p.(Leu1193*) | g.32472804A>T | [4] | DMD | 2 |
| 26 | c.3578T>G | p.(Leu1193*) | g.32472804A>C | [23] | DMD | 1 |
| 26 | c.3580C>T | p.(Gln1194*) | g.32472802G>A | [18] | DMD | 15 |
| 26 | c.3595G>T | p.(Glu1199*) | g.32472787C>A | [1] | ND | 1 |
| 27 | c.3622C>T | p.(Gln1208*) | g.32466737G>A | [3, 19] | DMD | 4 |
| 27 | c.3625C>T | p.(Gln1209*) | g.32466734G>A | [52] | DMD | 2 |
| 27 | c.3631G>T | p.[Glu1211*; Arg1202_1262del; Arg1202_1357del] | g.32466728C>A | [18] | BMD | 1 |
| 27 | c.3679C>T | p.(Gln1227*) | g.32466680G>A | [30] | DMD | 1 |
| 27 | c.3700G>T | p.(Glu1234*) | g.32466659C>A | [19] | BMD | 1 |
| 27 | c.3709A>T | p.(Lys1237*) | g.32466650T>A | [68] | DMD | 2 |
| 27 | c.3715G>T | p.(Glu1239*) | g.32466644C>A | [23] | DMD | 1 |
| 27 | c.3742C>T | p.(Gln1248*) | g.32466617G>A | [19] | OTHER | 2 |
| 27 | c.3746G>A | p.(Trp1249*) | g.32466613C>T | [1] | DMD | 1 |
| 27 | c.3747G>A | p.(Trp1249*) | g.32466612C>T | [69] | DMD | 1 |
| 27 | c.3769A>T | p.(Lys1257*) | g.32466590T>A | [3] | DMD | 1 |
| 27 | c.3774C>A | p.(Cys1258*) | g.32466585G>T | [7] | ND | 1 |
| 27 | c.3784G>T | p.(Glu1262*) | g.32466575C>A | [69] | DMD | 1 |
| 28 | c.3795G>A | p.(Trp1265*) | g.32459423C>T | [18] | DMD/BMD | 2 |
| 28 | c.3801T>A | p.(Cys1267*) | g.32459417A>T | [70] | OTHER | 1 |
| 28 | c.3804G>A | p.(Trp1268*) | g.32459414C>T | [19] | DMD | 1 |
| 28 | c.3818C>A | p.(Ser1273*) | g.32459400G>T | [20] | DMD | 1 |
| 28 | c.3822C>G | p.[Tyr1274*, Glu1263_Asp1307del, Glu1263_Glu1357del] | g.32459396G>C | [21, 33] | DMD | 5 |
| 28 | c.3822C>A | p.(Tyr1274*) | g.32459396G>T | [19] | DMD/BMD | 5 |
| 28 | c.3838A>T | p.(Lys1280*) | g.32459380T>A | [16] | DMD/BMD | 1 |
| 28 | c.3843G>A | p.(Trp1281*) | g.32459375C>T | [69] | BMD | 2 |
| 28 | c.3850G>T | p.[Glu1284*, Glu1263_Asp1307del, Glu1263_Glu1357del] | g.32459368C>A | [4] | BMD | 2 |
| 28 | c.3862A>T | p.(Lys1288*) | g.32459356T>A | [4] | DMD | 2 |
| 28 | c.3868A>T | p.(Lys1290*) | g.32459350T>A | [19] | DMD | 1 |
| 28 | c.3892G>T | p.(Gly1298*) | g.32459326C>A | [1] | OTHER | 1 |
| 29 | c.3923C>G | p.(Ser1308*) | g.32456506G>C | [1] | ND | 1 |
| 29 | c.3935T>A | p.[Leu1312*, Glu1263_Glu1357del, Ser1308_Glu1357del] | g.32456494A>T | [1] | BMD | 1 |
| 29 | c.3940C>T | p.[Arg1314*, Ser1308_Glu1357del, Glu1263_Glu1357del] | g.32456489G>A | [23] | BMD | 31 |
| 29 | c.3947C>A | p.(Ser1316*) | g.32456482G>T | [1] | DMD | 1 |
| 29 | c.3964C>T | p.(Gln1322*) | g.32456465G>A | [40] | ND | 1 |
| 29 | c.3982C>T | p.[Gln1328*, Glu1263_Asp1307del,Glu1263_Glu1357del] | g.32456447G>A | [19, 23] | DMD/BMD | 10 |
| 29 | c.4000G>T | p.[Gly1334*, Glu1263_Glu1357del, Ser1308_Glu1357del] | g.32456429C>A | [1] | BMD | 1 |
| 29 | c.4012G>T | p.(Glu1338*) | g.32456417C>A | [19] | BMD | 1 |
| 29 | c.4027G>T | p.(Glu1343*) | g.32456402C>A | [1] | ND | 3 |
| 29 | c.4033G>T | p.(Glu1345*) | g.32456396C>A | [1] | DMD | 2 |
| 29 | c.4057G>T | p.(Glu1353*) | g.32456372C>A | [48] | DMD | 3 |
| 29 | c.4066G>T | p.(Glu1356*) | g.32456363C>A | [13] | DMD | 2 |
| 30 | c.4084C>T | p.(Gln1362*) | g.32430018G>A | [8] | DMD | 2 |
| 30 | c.4087A>T | p.(Lys1363*) | g.32430015T>A | [12] | DMD | 1 |
| 30 | c.4099C>T | p.(Gln1367*) | g.32430003G>A | [10] | DMD | 3 |
| 30 | c.4108C>T | p.(Gln1370*) | g.32429994G>A | [25] | DMD | 7 |
| 30 | c.4117C>T | p.(Gln1373*) | g.32429985G>A | [3, 21, 22, 71] | DMD | 7 |
| 30 | c.4120G>T | p.(Glu1374*) | g.32429982C>A | [72] | DMD | 2 |
| 30 | c.4126G>T | p.(Glu1376*) | g.32429976C>A | [13] | DMD | 2 |
| 30 | c.4142T>G | p.(Leu1381*) | g.32429960A>C | [3] | DMD | 1 |
| 30 | c.4147C>T | p.(Gln1383*) | g.32429955G>A | [73] | DMD | 2 |
| 30 | c.4150G>T | p.(Glu1384*) | g.32429952C>A | [19, 56] | DMD | 2 |
| 30 | c.4174C>T | p.(Gln1392*) | g.32429928G>A | [21, 23, 53] | DMD | 5 |
| 30 | c.4213C>T | p.(Gln1405*) | g.32429889G>A | [11] | DMD/BMD | 2 |
| 30 | c.4222C>T | p.(Gln1408*) | g.32429880G>A | [16] | ND | 1 |
| 30 | c.4231C>T | p.(Gln1411*) | g.32429871G>A | [53, 65] | DMD | 2 |
| 31 | c.4240C>T | p.(Gln1414*) | g.32408292G>A | [19] | BMD | 1 |
| 31 | c.4250T>A | p.[Leu1417*; Lys1412_Gln1448del] | g.32408282A>T | [74] | BMD | 4 |
| 31 | c.4285A>T | p.(Lys1429*) | g.32408247T>A | [19] | BMD | 1 |
| 31 | c.4294C>T | p.[Gln1432*,Ile1413_Lys1449del] | g.32408238G>A | [38] | BMD | 3 |
| 31 | c.4303G>T | p.[Glu1435*, Lys1412_Gln1448del] | g.32408229C>A | [18] | DMD/BMD | 1 |
| 31 | c.4312C>T | p.(Gln1438*) | g.32408220G>A | [16] | ND | 1 |
| 31 | c.4315A>T | p.(Arg1439*) | g.32408217T>A | [16] | DMD/BMD | 1 |
| 32 | c.4352T>A | p.(Leu1451*) | g.32407784A>T | [19] | DMD | 1 |
| 32 | c.4354C>T | p.(Gln1452*) | g.32407782G>A | [1] | DMD | 1 |
| 32 | c.4375C>T | p.(Arg1459*) | g.32407761G>A | [3, 13, 21] | DMD | 14 |
| 32 | c.4384C>T | p.(Gln1462*) | g.32407752G>A | [10] | DMD | 3 |
| 32 | c.4405C>T | p.(Gln1469*) | g.32407731G>A | [30] | DMD | 7 |
| 32 | c.4414C>T | p.(Gln1472*) | g.32407722G>A | [11] | DMD | 7 |
| 32 | c.4480G>T | p.(Glu1494*) | g.32407656C>A | [11] | DMD | 1 |
| 32 | c.4483C>T | p.(Gln1495*) | g.32407653G>A | [3, 19, 37] | DMD | 5 |
| 32 | c.4486G>T | p.(Glu1496*) | g.32407650C>A | [26, 75] | DMD | 2 |
| 32 | c.4495C>T | p.(Gln1499*) | g.32407641G>A | [13] | DMD | 3 |
| 32 | c.4499C>A | p.(Ser1500*) | g.32407637G>T | [21] | DMD | 1 |
| 32 | c.4501C>T | p.(Gln1501*) | g.32407635G>A | [13] | DMD | 2 |
| 33 | c.4527T>A | p.(Tyr1509*) | g.32404574A>T | [1] | DMD | 1 |
| 33 | c.4527T>G | p.(Tyr1509*) | g.32404574A>C | [18] | DMD | 4 |
| 33 | c.4540G>T | p.(Glu1514*) | g.32404561C>A | [13] | DMD | 1 |
| 33 | c.4558G>T | p.(Glu1520*) | g.32404543C>A | [1] | DMD | 1 |
| 33 | c.4559G>T | p.(Glu1520*) | g.32404542C>A | [76] | DMD | 1 |
| 33 | c.4570A>T | p.(Lys1524*) | g.32404531T>A | [27] | DMD/BMD | 1 |
| 33 | c.4576G>T | p.(Gly1526*) | g.32404525C>A | [1] | BMD | 1 |
| 33 | c.4582C>T | p.(Gln1528*) | g.32404519G>A | [3, 23] | DMD | 2 |
| 33 | c.4591C>T | p.(Gln1531*) | g.32404510G>A | [15] | OTHER | 3 |
| 33 | c.4600C>T | p.(Gln1534*) | g.32404501G>A | [54] | DMD | 7 |
| 33 | c.4606G>T | p.(Glu1536*) | g.32404495C>A | [37] | DMD | 2 |
| 33 | c.4618G>T | p.(Glu1540*) | g.32404483C>A | [19] | DMD | 4 |
| 33 | c.4660G>T | p.(Glu1554*) | g.32404441C>A | [3] | DMD | 1 |
| 33 | c.4666G>T | p.(Gly1556*) | g.32404435C>A | [18] | DMD | 3 |
| 34 | c.4687A>T | p.(Lys1563*) | g.32398785T>A | [18] | DMD | 1 |
| 34 | c.4690C>T | p.(Gln1564*) | g.32398782G>A | [52] | DMD | 1 |
| 34 | c.4693C>T | p.(Gln1565*) | g.32398779G>A | [30] | DMD | 4 |
| 34 | c.4697T>A | p.(Leu1566*) | g.32398775A>T | [19] | DMD | 1 |
| 34 | c.4702A>T | p.(Lys1568*) | g.32398770T>A | [1] | DMD | 1 |
| 34 | c.4711A>T | p.(Lys1571*) | g.32398761T>A | [25] | DMD | 4 |
| 34 | c.4729C>T | p.(Arg1577*) | g.32398743G>A | [10] | DMD | 31 |
| 34 | c.4732A>T | p.(Lys1578*) | g.32398740T>A | [23] | DMD | 2 |
| 34 | c.4735G>T | p.(Glu1579*) | g.32398737C>A | [1] | DMD/BMD | 1 |
| 34 | c.4757G>A | p.(Trp1586*) | g.32398715C>T | [18] | DMD | 1 |
| 34 | c.4758G>A | p.(Trp1586*) | g.32398714C>T | [54] | DMD | 1 |
| 34 | c.4777G>T | p.(Glu1593*) | g.32398695C>A | [39] | DMD | 1 |
| 34 | c.4781T>A | p.(Leu1574*) | g.32398691A>T | [29] | DMD | 1 |
| 34 | c.4793C>A | p.(Ser1598*) | g.32398679G>T | [19] | DMD | 1 |
| 34 | c.4838G>A | p.(Trp1613*) | g.32398634C>T | [4, 56] | DMD | 3 |
| 34 | c.4843A>T | p.(Lys1615*) | g.32398629T>A | [1] | DMD/BMD | 1 |
| 35 | c.4852C>T | p.(Gln1618*) | g.32383310G>A | [1] | DMD | 3 |
| 35 | c.4858G>T | p.(Glu1620*) | g.32383304C>A | [7] | DMD/BMD | 1 |
| 35 | c.4864G>T | p.(Glu1622*) | g.32383298C>A | [19] | DMD | 1 |
| 35 | c.4870C>T | p.(Gln1624*) | g.32383292G>A | [30] | DMD | 8 |
| 35 | c.4915A>T | p.(Lys1639*) | g.32383247T>A | [69] | DMD | 2 |
| 35 | c.4933A>T | p.(Lys1645*) | g.32383229T>A | [55] | DMD | 1 |
| 35 | c.4948G>T | p.(Glu1650*) | g.32383214C>A | [1] | ND | 1 |
| 35 | c.4954A>T | p.(Lys1652*) | g.32383208T>A | [63] | DMD | 2 |
| 35 | c.4979G>A | p.(Trp1660*) | g.32383183C>T | [52] | BMD | 1 |
| 35 | c.4980G>A | p.(Trp1660*) | g.32383182C>T | [1] | BMD | 1 |
| 35 | c.4996C>T | p.(Arg1666*) | g.32383166G>A | [30] | DMD | 30 |
| 35 | c.5002G>T | p.(Glu1668*) | g.32383160C>A | [21] | BMD | 1 |
| 35 | c.5009G>A | p.(Trp1670*) | g.32383153C>T | [34] | DMD | 1 |
| 35 | c.5021T>A | p.(Leu1674*) | g.32383141A>T | [1] | DMD/BMD | 1 |
| 35 | c.5024T>A | p.(Leu1675*) | g.32383138A>T | [16] | ND | 1 |
| 36 | c.5031C>A | p.(Tyr1677*) | g.32382822G>T | [10] | DMD | 1 |
| 36 | c.5032C>T | p.(Gln1678*) | g.32382821G>A | [30] | DMD | 1 |
| 36 | c.5044G>T | p.(Glu1682*) | g.32382809C>A | [13, 18, 67] | DMD | 3 |
| 36 | c.5082G>A | p.(Trp1694*) | g.32382771C>T | [13] | DMD | 2 |
| 36 | c.5089C>T | p.(Gln1697*) | g.32382764G>A | [1] | DMD | 2 |
| 36 | c.5110G>T | p.(Glu1704*) | g.32382743C>A | [16] | ND | 1 |
| 36 | c.5119A>T | p.(Lys1707*) | g.32382734T>A | [3] | DMD | 1 |
| 36 | c.5131C>T | p.(Gln1711*) | g.32382722G>A | [37] | DMD | 5 |
| 36 | c.5134C>T | p.(Gln1712*) | g.32382719G>A | [19] | DMD | 4 |
| 36 | c.5140G>T | p.(Glu1714*) | g.32382713C>A | [19] | DMD | 3 |
| 37 | c.5159T>A | p.(Leu1720*) | g.32381071A>T | [66] | DMD | 1 |
| 37 | c.5167G>T | p.(Glu1723*) | g.32381063C>A | [21, 69] | DMD | 3 |
| 37 | c.5188A>T | p.(Lys1730*) | g.32381042T>A | [1] | DMD | 1 |
| 37 | c.5209C>T | p.(Gln1737*) | g.32381021G>A | [22, 71] | DMD | 4 |
| 37 | c.5255T>G | p.(Leu1752*) | g.32380975A>C | [19] | DMD | 2 |
| 37 | c.5260G>T | p.(Glu1754*) | g.32380970C>A | [77] | BMD | 1 |
| 37 | c.5266C>T | p.(Gln1756*) | g.32380964G>A | [34] | DMD | 3 |
| 37 | c.5287C>T | p.[Arg1763*, Arg1719_Lys1775del] | g.32380943G>A | [25] | BMD | 22 |
| 37 | c.5308A>T | p.(Arg1770*) | g.32380922T>A | [1] | DMD | 1 |
| 37 | c.5314A>T | p.(Lys1772*) | g.32380916T>A | [39] | DMD | 1 |
| 38 | c.5341A>T | p.(Lys1781*) | g.32366630T>A | [66] | DMD/BMD | 3 |
| 38 | c.5344G>T | p.(Glu1782*) | g.32366627C>A | [19] | DMD | 2 |
| 38 | c.5350G>T | p.(Glu1784*) | g.32366621C>A | [18] | DMD | 2 |
| 38 | c.5353C>T | p.(Gln1785*) | g.32366618G>A | [23] | DMD/BMD | 2 |
| 38 | c.5363C>G | p.(Ser1788*) | g.32366608G>C | [16] | DMD/BMD | 1 |
| 38 | c.5371C>T | p.[Gln1791*,Ala1776_Met1816del] | g.32366600G>A | [13, 38] | DMD/BMD | 11 |
| 38 | c.5398G>T | p.(Glu1800*) | g.32366573C>A | [19] | BMD | 3 |
| 38 | c.5404C>T | p.(Gln1802*) | g.32366567G>A | [78] | BMD | 5 |
| 38 | c.5407C>T | p.[Gln1803*, Ala1776_Met1816del] | g.32366564G>A | [13, 38] | BMD | 5 |
| 39 | c.5452G>T | p.(Glu1818*) | g.32364194C>A | [1] | BMD | 1 |
| 39 | c.5461G>T | p.(Glu1821*) | g.32364185C>A | [12] | DMD | 1 |
| 39 | c.5476G>T | p.(Glu1826*) | g.32364170C>A | [22, 54] | DMD | 3 |
| 39 | c.5480T>A | p.[Leu1827*, Ala1776_Lys1862del, Asn1817_Lys1862del] | g.32364166A>T | [1] | BMD | 2 |
| 39 | c.5485C>T | p.(Gln1829*) | g.32364161G>A | [49] | DMD | 1 |
| 39 | c.5488A>T | p.(Arg1830*) | g.32364158T>A | [9] | DMD/BMD | 5 |
| 39 | c.5503C>T | p.(Gln1835*) | g.32364143G>A | [13] | DMD | 1 |
| 39 | c.5506C>T | p.(Gln1836*) | g.32364140G>A | [16] | ND | 1 |
| 39 | c.5521G>T | p.(Glu1841*) | g.32364125C>A | [37] | ND | 1 |
| 39 | c.5530C>T | p.(Arg1844*) | g.32364116G>A | [3, 13, 19, 30] | DMD | 28 |
| 39 | c.5533G>T | p.(Glu1845*) | g.32364113C>A | [79] | OTHER | 1 |
| 39 | c.5542A>T | p.(Lys1848*) | g.32364104T>A | [30] | DMD | 1 |
| 39 | c.5551C>T | p.[Gln1851*; Asn1817_Lys1862del] | g.32364095G>A | [58] | DMD | 7 |
| 39 | c.5554C>T | p.(Gln1852*) | g.32364092G>A | [15, 69] | OTHER | 4 |
| 39 | c.5561T>G | p.(Leu1854*) | g.32364085A>C | [1] | ND | 1 |
| 39 | c.5563C>T | p.(Gln1855*) | g.32364083G>A | [54] | DMD | 7 |
| 40 | c.5599C>T | p.(Gln1867*) | g.32361391G>A | [13] | DMD | 1 |
| 40 | c.5602A>T | p.(Arg1868*) | g.32361388T>A | [13] | DMD | 1 |
| 40 | c.5608A>T | p.(Lys1870*) | g.32361382T>A | [19] | DMD | 3 |
| 40 | c.5611A>T | p.(Lys1871*) | g.32361379T>A | [4, 20] | DMD | 4 |
| 40 | c.5632C>T | p.(Gln1878*) | g.32361358G>A | [21, 53] | OTHER | 4 |
| 40 | c.5637G>A | p.(Trp1879*) | g.32361353C>T | [25] | DMD | 3 |
| 40 | c.5640T>A | p.(Tyr1880*) | g.32361350A>T | [1] | DMD/BMD | 2 |
| 40 | c.5641C>T | p.(Gln1881*) | g.32361349G>A | [30] | DMD | 2 |
| 40 | c.5646C>A | p.(Tyr1882*) | g.32361344G>T | [19] | DMD | 3 |
| 40 | c.5646C>G | p.(Tyr1882*) | g.32361344G>C | [56] | DMD | 1 |
| 40 | c.5647A>T | p.(Lys1883*) | g.32361343T>A | [16] | ND | 1 |
| 40 | c.5653C>T | p.(Gln1885*) | g.32361337G>A | [10] | DMD | 3 |
| 40 | c.5671A>T | p.(Lys1891*) | g.32361319T>A | [1] | DMD/BMD | 2 |
| 40 | c.5699T>G | p.(Leu1900*) | g.32361291A>C | [1] | DMD/BMD | 2 |
| 40 | c.5713G>T | p.(Glu1905*) | g.32361277C>A | [16] | ND | 1 |
| 40 | c.5725G>T | p.(Glu1909*) | g.32361265C>A | [19] | BMD | 1 |
| 41 | c.5740G>T | p.(Glu1914*) | g.32360399C>A | [1] | ND | 1 |
| 41 | c.5752G>T | p.(Glu1918*) | g.32360387C>A | [19] | DMD/BMD | 3 |
| 41 | c.5758C>T | p.(Gln1920*) | g.32360381G>A | [19] | DMD | 5 |
| 41 | c.5761A>T | p.(Lys1921*) | g.32360378T>A | [69] | DMD | 1 |
| 41 | c.5764A>T | p.(Lys1922*) | g.32360375T>A | [7] | DMD/BMD | 1 |
| 41 | c.5770G>T | p.(Glu1924*) | g.32360369C>A | [13] | DMD | 2 |
| 41 | c.5773G>T | p.(Glu1925*) | g.32360366C>A | [54] | DMD/BMD | 6 |
| 41 | c.5800G>T | p.(Glu1934*) | g.32360339C>A | [30] | DMD | 2 |
| 41 | c.5807T>A | p.(Leu1936*) | g.32360332A>T | [1] | DMD/BMD | 1 |
| 41 | c.5835G>T | p.(Glu1946*) | g.32360304C>A | [1] | BMD | 1 |
| 41 | c.5845C>T | p.(Gln1949*) | g.32360294G>A | [1] | ND | 1 |
| 41 | c.5851C>T | p.(Gln1951*) | g.32360288G>A | [3, 20] | DMD | 6 |
| 41 | c.5867G>A | p.(Trp1956*) | g.32360272C>T | [19] | DMD | 1 |
| 41 | c.5868G>A | p.(Trp1956*) | g.32360271C>T | [21] | ND | 1 |
| 41 | c.5872G>T | p.(Glu1958*) | g.32360267C>A | [16] | ND | 1 |
| 41 | c.5878G>T | p.(Glu1960*) | g.32360261C>A | [37] | DMD | 3 |
| 41 | c.5893C>T | p.(Gln1965*) | g.32360246G>A | [79] | OTHER | 2 |
| 41 | c.5899C>T | p.[Arg1967*,Glu1914_Ile1974del] | g.32360240G>A | [38] | DMD | 35 |
| 41 | c.5902A>T | p.(Arg1968*) | g.32360237T>A | [19] | DMD | 1 |
| 41 | c.5917C>T | p.(Gln1973*) | g.32360222G>A | [19, 46] | DMD | 2 |
| 42 | c.5938G>T | p.(Glu1980*) | g.32328378C>A | [37] | DMD/BMD | 2 |
| 42 | c.5985T>G | p.[Tyr1995*, His1975_Tyr1995del] | g.32328331A>C | [13, 18] | DMD | 3 |
| 42 | c.6000T>A | p.(Tyr2000*) | g.32328316A>T | [16] | DMD/BMD | 1 |
| 42 | c.6007G>T | p.(Glu2003*) | g.32328309C>A | [21] | ND | 1 |
| 42 | c.6023C>G | p.(Ser2008*) | g.32328293G>C | [23] | DMD | 1 |
| 42 | c.6023C>A | p.(Ser2008*) | g.32328293G>T | [52] | DMD | 1 |
| 42 | c.6035T>G | p.(Leu2012*) | g.32328281A>C | [7] | DMD/BMD | 1 |
| 42 | c.6037G>T | p.(Glu2013*) | g.32328279C>A | [19] | DMD | 1 |
| 42 | c.6072T>A | p.(Cys2024*) | g.32328244A>T | [54] | DMD | 1 |
| 42 | c.6085G>T | p.(Glu2029*) | g.32328231C>A | [69] | DMD | 1 |
| 42 | c.6103G>T | p.(Glu2035*) | g.32328213C>A | [19] | DMD | 3 |
| 42 | c.6106G>T | p.(Glu2036*) | g.32328210C>A | [23] | DMD | 1 |
| 43 | c.6136C>T | p.(Gln2046*) | g.32305800G>A | [13] | DMD | 1 |
| 43 | c.6139C>T | p.(Gln2047*) | g.32305797G>A | [1] | ND | 1 |
| 43 | c.6208G>T | p.(Glu2070*) | g.32305728C>A | [15] | DMD | 2 |
| 43 | c.6223C>T | p.(Gln2075*) | g.32305713G>A | [28] | DMD | 2 |
| 43 | c.6226G>T | p.(Glu2076*) | g.32305710C>A | [1] | DMD/BMD | 1 |
| 43 | c.6238C>T | p.(Gln2080*) | g.32305698G>A | [25] | DMD | 2 |
| 43 | c.6250C>T | p.(Gln2084*) | g.32305686G>A | [37] | ND | 1 |
| 43 | c.6255G>A | p.(Trp2085*) | g.32305681C>T | [19] | DMD | 5 |
| 43 | c.6256G>T | p.(Glu2086*) | g.32305680C>A | [3] | DMD | 1 |
| 43 | c.6259A>T | p.(Lys2087*) | g.32305677T>A | [80] | DMD | 1 |
| 43 | c.6276C>G | p.(Tyr2092*) | g.32305660G>C | [39] | DMD | 1 |
| 43 | c.6277A>T | p.(Lys2093*) | g.32305659T>A | [71] | ND | 2 |
| 43 | c.6283C>T | p.(Arg2095*) | g.32305653G>A | [10] | DMD | 27 |
| 43 | c.6286C>T | p.(Gln2096*) | g.32305650G>A | [4] | DMD | 1 |
| 44 | c.6292C>T | p.(Arg2098*) | g.32235179G>A | [10] | DMD | 27 |
| 44 | c.6310G>T | p.(Glu2104*) | g.32235161C>A | [3] | DMD | 2 |
| 44 | c.6317G>A | p.(Trp2106*) | g.32235154C>T | [16] | ND | 1 |
| 44 | c.6318G>A | p.(Trp2106*) | g.32235153C>T | [9, 19] | DMD/BMD | 3 |
| 44 | c.6340A>T | p.(Lys2114*) | g.32235131T>A | [1] | DMD/BMD | 1 |
| 44 | c.6352C>T | p.(Gln2118*) | g.32235119G>A | [19] | DMD | 4 |
| 44 | c.6356G>A | p.(Trp2119*) | g.32235115C>T | [13] | DMD | 1 |
| 44 | c.6364G>T | p.(Glu2122*) | g.32235107C>A | [25] | DMD | 1 |
| 44 | c.6370G>T | p.(Glu2124*) | g.32235101C>A | [3] | DMD | 1 |
| 44 | c.6373C>T | p.(Gln2125*) | g.32235098G>A | [3] | DMD | 4 |
| 44 | c.6382A>T | p.(Arg2128*) | g.32235089T>A | [1] | DMD | 1 |
| 44 | c.6391C>T | p.(Gln2131*) | g.32235080G>A | [19] | DMD | 2 |
| 44 | c.6407G>A | p.(Trp2136*) | g.32235064C>T | [1] | ND | 1 |
| 44 | c.6408G>A | p.(Trp2136*) | g.32235063C>T | [7] | DMD/BMD | 1 |
| 44 | c.6423C>A | p.(Tyr2141*) | g.32235048G>T | [27] | DMD | 5 |
| 44 | c.6423C>G | p.(Tyr2141*) | g.32235048G>C | [23] | DMD | 2 |
| 44 | c.6424A>T | p.(Lys2142*) | g.32235047T>A | [16] | DMD | 1 |
| 44 | c.6428G>A | p.(Trp2143*) | g.32235043C>T | [1] | ND | 1 |
| 44 | c.6429G>A | p.(Trp2143*) | g.32235042C>T | [28] | DMD | 1 |
| 44 | c.6432T>A | p.(Tyr2144*) | g.32235039A>T | [18] | DMD | 2 |
| 44 | c.6436A>T | p.(Lys2146*) | g.32235035T>A | [12] | DMD | 3 |
| 44 | c.6439G>T | p.(Gln2147*) | g.31986631C>A | [81] | ND | 1 |
| 45 | c.6445C>T | p.(Gln2149*) | g.31986625G>A | [19] | DMD | 1 |
| 45 | c.6460C>T | p.(Gln2154*) | g.31986610G>A | [12] | DMD | 4 |
| 45 | c.6499G>T | p.(Glu2167*) | g.31986571C>A | [1] | DMD | 1 |
| 45 | c.6502G>T | p.(Glu2168*) | g.31986568C>A | [1] | ND | 1 |
| 45 | c.6544C>T | p.(Gln2182*) | g.31986526G>A | [19, 55] | DMD | 6 |
| 45 | c.6550A>T | p.(Lys2184*) | g.31986520T>A | [44, 59] | ND | 1 |
| 45 | c.6576G>A | p.(Trp2192*) | g.31986494C>T | [54] | DMD | 1 |
| 45 | c.6577C>T | p.(Gln2193*) | g.31986493G>A | [1] | DMD | 1 |
| 45 | c.6592C>T | p.(Gln2198*) | g.31986478G>A | [19] | DMD/BMD | 2 |
| 45 | c.6607A>T | p.(Lys2203*) | g.31986463T>A | [23] | DMD | 1 |
| 46 | c.6641C>A | p.(Ser2214*) | g.31950318G>T | [19] | DMD | 1 |
| 46 | c.6649C>T | p.(Gln2217*) | g.31950310G>A | [19] | DMD | 2 |
| 46 | c.6659T>G | p.(Leu2220*) | g.31950300A>C | [13] | DMD | 1 |
| 46 | c.6674T>A | p.(Leu2225*) | g.31950285A>T | [54] | DMD | 1 |
| 46 | c.6674T>G | p.(Leu2225*) | g.31950285A>C | [28] | DMD | 1 |
| 46 | c.6677G>A | p.(Trp2226*) | g.31950282C>T | [1] | ND | 2 |
| 46 | c.6678G>A | p.(Trp2226*) | g.31950281C>T | [52] | DMD | 3 |
| 46 | c.6721G>T | p.(Gly2241*) | g.31950238C>A | [19] | DMD/BMD | 1 |
| 46 | c.6730C>T | p.(Gln2244*) | g.31950229G>A | [15] | DMD | 1 |
| 46 | c.6733C>T | p.(Gln2245*) | g.31950226G>A | [1] | DMD | 1 |
| 46 | c.6742G>T | p.(Glu2248*) | g.31950217C>A | [25] | DMD | 1 |
| 46 | c.6754C>T | p.(Gln2252*) | g.31950205G>A | [23] | DMD | 1 |
| 47 | c.6790C>T | p.(Gln2264*) | g.31947835G>A | [11] | DMD | 2 |
| 47 | c.6805C>T | p.(Gln2269*) | g.31947820G>A | [18] | DMD | 2 |
| 47 | c.6805C>A | p.(Gln2269*) | g.31947820G>T | [18] | DMD | 1 |
| 47 | c.6809T>G | p.(Leu2270*) | g.31947816A>C | [82] | DMD | 1 |
| 47 | c.6856G>T | p.(Glu2286*) | g.31947769C>A | [19] | DMD | 2 |
| 47 | c.6862C>T | p.(Gln2288*) | g.31947763G>A | [18] | DMD | 1 |
| 47 | c.6868A>T | p.(Lys2290*) | g.31947757T>A | [19] | DMD | 1 |
| 47 | c.6880A>T | p.(Lys2294*) | g.31947745T>A | [39] | DMD | 1 |
| 47 | c.6889C>T | p.(Gln2297*) | g.31947736G>A | [1] | DMD | 1 |
| 47 | c.6905G>A | p.(Trp2302*) | g.31947720C>T | [10] | DMD | 6 |
| 47 | c.6906G>A | p.(Trp2302*) | g.31947719C>T | [19] | DMD/BMD | 2 |
| 48 | c.6926T>A | p.(Leu2309*) | g.31893477A>T | [1] | DMD | 1 |
| 48 | c.6943G>T | p.(Glu2315*) | g.31893460C>A | [71] | DMD/BMD | 7 |
| 48 | c.6949G>T | p.(Glu2317*) | g.31893454C>A | [13] | DMD | 1 |
| 48 | c.6955C>T | p.(Gln2319*) | g.31893448G>A | [83] | DMD | 1 |
| 48 | c.6973C>T | p.(Gln2325*) | g.31893430G>A | [13, 50] | DMD | 3 |
| 48 | c.6975C>T | p.(Gln2325*) | g.31893428G>A | [13] | DMD | 1 |
| 48 | c.6979G>T | p.(Glu2327*) | g.31893424C>A | [1] | ND | 1 |
| 48 | c.6982A>T | p.(Lys2328*) | g.31893421T>A | [16] | DMD/BMD | 1 |
| 48 | c.7006C>T | p.(Gln2336*) | g.31893397G>A | [33] | DMD/BMD | 3 |
| 48 | c.7010T>G | p.(Leu2337*) | g.31893393A>C | [1] | DMD | 1 |
| 48 | c.7029G>A | p.(Trp2343*) | g.31893374C>T | [44] | DMD | 2 |
| 48 | c.7031T>A | p.(Leu2344*) | g.31893372A>T | [36] | ND | 1 |
| 48 | c.7054G>T | p.(Glu2352*) | g.31893349C>A | [12] | DMD | 1 |
| 48 | c.7075C>T | p.(Gln2359*) | g.31893328G>A | [19] | DMD | 1 |
| 49 | c.7105G>T | p.[Glu2369*, Glu2367_Lys2400del] | g.31854930C>A | [25] | BMD | 5 |
| 49 | c.7126C>T | p.(Gln2376*) | g.31854909G>A | [13] | DMD | 1 |
| 49 | c.7159C>T | p.[Gln2387*, Glu2367_Lys2400del] | g.31854876G>A | [84] | ND | 2 |
| 49 | c.7170C>G | p.(Tyr2390*) | g.31854865G>C | [16] | ND | 1 |
| 49 | c.7189C>T | p.(Gln2397*) | g.31854846G>A | [16] | DMD/BMD | 1 |
| 50 | c.7204A>T | p.(Lys2402*) | g.31838197T>A | [1] | ND | 1 |
| 50 | c.7229G>A | p.(Trp2410*) | g.31838172C>T | [1] | DMD/BMD | 1 |
| 50 | c.7255G>T | p.(Glu2419*) | g.31838146C>A | [18] | DMD | 2 |
| 50 | c.7288G>T | p.(Gly2430*) | g.31838113C>A | [3] | DMD | 1 |
| 51 | c.7318C>T | p.(Gln2440*) | g.31792301G>A | [85] | OTHER | 1 |
| 51 | c.7339C>T | p.(Gln2447*) | g.31792280G>A | [12] | DMD | 1 |
| 51 | c.7402G>T | p.(Glu2468*) | g.31792217C>A | [42] | DMD | 1 |
| 51 | c.7436G>A | p.(Trp2479*) | g.31792183C>T | [1] | DMD | 1 |
| 51 | c.7471C>T | p.(Glu2491*) | g.31792148G>A | [1] | DMD | 1 |
| 51 | c.7510G>T | p.(Glu2504*) | g.31792109C>A | [49] | DMD | 1 |
| 51 | c.7537C>T | p.(Gln2513*) | g.31792082G>A | [86] | ND | 1 |
| 51 | c.7561G>T | p.(Glu2521*) | g.31747847C>A | [37] | ND | 1 |
| 52 | c.7564C>T | p.(Gln2522*) | g.31747844G>A | [87] | DMD | 2 |
| 52 | c.7576C>T | p.(Gln2526*) | g.31747832G>A | [19] | DMD | 2 |
| 52 | c.7582G>T | p.(Glu2528*) | g.31747826C>A | [28] | DMD | 1 |
| 52 | c.7585G>T | p.(Glu2529*) | g.31747823C>A | [20] | DMD | 2 |
| 52 | c.7657C>T | p.(Arg2553*) | g.31747751G>A | [10] | DMD | 33 |
| 53 | c.7672C>T | p.(Gln2558*) | g.31697692G>A | [30] | DMD | 2 |
| 53 | c.7682G>A | p.(Trp2561*) | g.31697682C>T | [19] | DMD | 1 |
| 53 | c.7683G>A | p.(Trp2561*) | g.31697681C>T | [1] | DMD/BMD | 1 |
| 53 | c.7693C>T | p.(Gln2565*) | g.31697671G>A | [19] | DMD | 2 |
| 53 | c.7696G>T | p.(Glu2566*) | g.31697668C>A | [1] | DMD | 1 |
| 53 | c.7717C>T | p.(Gln2573*) | g.31697647G>A | [7] | DMD/BMD | 1 |
| 53 | c.7720C>T | p.(Gln2574*) | g.31697644G>A | [19] | DMD | 1 |
| 53 | c.7736T>A | p.(Leu2579*) | g.31697628A>T | [1] | DMD | 4 |
| 53 | c.7736T>G | p.(Leu2579*) | g.31697628A>C | [1] | DMD | 1 |
| 53 | c.7750C>T | p.(Gln2584*) | g.31697614G>A | [13] | DMD | 1 |
| 53 | c.7755G>A | p.(Trp2585*) | g.31697609C>T | [27] | DMD | 1 |
| 53 | c.7771G>T | p.(Glu2591*) | g.31697593C>A | [1] | DMD/BMD | 1 |
| 53 | c.7792C>T | p.(Gln2598*) | g.31697572G>A | [21] | ND | 2 |
| 53 | c.7798A>T | p.(Arg2600*) | g.31697566T>A | [1] | DMD/BMD | 1 |
| 53 | c.7814C>G | p.(Ser2605*) | g.31697550G>C | [19] | DMD | 2 |
| 53 | c.7817G>A | p.(Trp2606*) | g.31697547C>T | [18] | DMD | 1 |
| 53 | c.7818G>A | p.(Trp2606*) | g.31697546C>T | [1] | DMD/BMD | 1 |
| 53 | c.7822G>T | p.(Glu2608*) | g.31697542C>A | [19] | DMD | 2 |
| 53 | c.7855A>T | p.(Lys2619*) | g.31697509T>A | [18] | DMD | 1 |
| 53 | c.7864G>T | p.(Glu2622*) | g.31697500C>A | [7] | DMD/BMD | 1 |
| 54 | c.7873C>T | p.(Gln2625*) | g.31676261G>A | [13] | DMD | 1 |
| 54 | c.7894C>T | p.(Gln2632*) | g.31676240G>A | [13] | DMD | 3 |
| 54 | c.7898G>A | p.(Trp2633*) | g.31676236C>T | [80] | DMD | 1 |
| 54 | c.7899G>A | p.(Trp2633*) | g.31676235C>T | [43] | ND | 1 |
| 54 | c.7969A>T | p.(Arg2657*) | g.31676165T>A | [23] | DMD | 1 |
| 54 | c.8009G>A | p.(Trp2670*) | g.31676125C>T | [63] | DMD | 4 |
| 54 | c.8010G>A | p.(Trp2670*) | g.31676124C>T | [13] | DMD | 3 |
| 55 | c.8038C>T | p.(Arg2680*) | g.31645969G>A | [3, 53, 71] | DMD | 18 |
| 55 | c.8053G>T | p.(Glu2685*) | g.31645954C>A | [13] | DMD | 2 |
| 55 | c.8069T>G | p.(Leu2690*) | g.31645938A>C | [69] | DMD | 5 |
| 55 | c.8074C>T | p.(Gln2692*) | g.31645933G>A | [28] | DMD | 2 |
| 55 | c.8077C>T | p.(Gln2693*) | g.31645930G>A | [1] | DMD | 1 |
| 55 | c.8098A>T | p.(Lys2700*) | g.31645909T>A | [45] | DMD | 1 |
| 55 | c.8146C>T | p.(Gln2716*) | g.31645861G>A | [19] | DMD | 1 |
| 55 | c.8161A>T | p.(Lys2721*) | g.31645846T>A | [19] | DMD | 2 |
| 55 | c.8176G>T | p.(Glu2726*) | g.31645831C>A | [39] | DMD | 1 |
| 55 | c.8194A>T | p.(Lys2732*) | g.31645813T>A | [3] | DMD | 2 |
| 55 | c.8197G>T | p.(Gln2733*) | g.31645810C>A | [44] | ND | 1 |
| 55 | c.8209C>T | p.(Gln2737*) | g.31645798G>A | [40] | ND | 1 |
| 55 | c.8213G>A | p.(Trp2738*) | g.31645794C>T | [37] | DMD | 1 |
| 55 | c.8214G>A | p.(Trp2738*) | g.31645793C>T | [4] | DMD | 5 |
| 55 | c.8215C>T | p.(Gln2739*) | g.31645792G>A | [3] | DMD/BMD | 2 |
| 56 | c.8269G>T | p.(Glu2757*) | g.31525519C>A | [55] | DMD | 1 |
| 56 | c.8299G>T | p.(Glu2767*) | g.31525489C>A | [13] | DMD | 2 |
| 56 | c.8326C>T | p.(Gln2776*) | g.31525462G>A | [1] | DMD | 1 |
| 56 | c.8353A>T | p.(Lys2785*) | g.31525435T>A | [19] | BMD | 1 |
| 56 | c.8357G>A | p.(Trp2786*) | g.31525431C>T | [45] | DMD | 2 |
| 56 | c.8358G>A | p.(Trp2786*) | g.31525430C>T | [1] | DMD/BMD | 1 |
| 56 | c.8371A>T | p.(Lys2791*) | g.31525417T>A | [34] | DMD | 2 |
| 56 | c.8374A>T | p.(Lys2792*) | g.31525414T>A | [10] | DMD | 1 |
| 57 | c.8416C>T | p.(Gln2806*) | g.31515036G>A | [13, 28] | DMD | 4 |
| 57 | c.8420G>A | p.(Trp2807*) | g.31515032C>T | [25] | DMD | 4 |
| 57 | c.8422A>T | p.(Lys2808*) | g.31515030T>A | [52] | DMD | 1 |
| 57 | c.8443C>T | p.(Gln2815*) | g.31515009G>A | [3, 55] | DMD | 5 |
| 57 | c.8459G>A | p.(Trp2820*) | g.31514993C>T | [13] | DMD | 1 |
| 57 | c.8460G>A | p.(Trp2820*) | g.31514992C>T | [19] | DMD | 3 |
| 57 | c.8464C>T | p.(Gln2822*) | g.31514988G>A | [37] | DMD | 3 |
| 57 | c.8479G>T | p.(Glu2827*) | g.31514973C>A | [13] | DMD | 1 |
| 57 | c.8483T>G | p.(Leu2828*) | g.31514969A>C | [37] | ND | 1 |
| 57 | c.8491C>T | p.(Gln2831*) | g.31514961G>A | [19] | DMD | 1 |
| 57 | c.8527A>T | p.(Lys2843*) | g.31514925T>A | [19] | DMD | 1 |
| 58 | c.8560G>T | p.(Glu2854*) | g.31497208C>A | [13] | DMD | 1 |
| 58 | c.8575G>T | p.(Glu2859*) | g.31497193C>A | [21] | DMD | 2 |
| 58 | c.8608C>T | p.(Arg2870*) | g.31497160G>A | [10] | DMD | 45 |
| 58 | c.8626C>T | p.(Gln2876*) | g.31497142G>A | [13] | DMD | 1 |
| 58 | c.8647A>T | p.(Lys2883*) | g.31497121T>A | [16] | ND | 1 |
| 58 | c.8655C>A | p.(Tyr2885*) | g.31497113G>T | [1] | OTHER | 1 |
| 58 | c.8656C>T | p.(Gln2886*) | g.31497112G>A | [88] | DMD | 1 |
| 59 | c.8680G>T | p.(Glu2894*) | g.31496480C>A | [19] | DMD/BMD | 1 |
| 59 | c.8686A>T | p.(Arg2896*) | g.31496474T>A | [12] | DMD/BMD | 1 |
| 59 | c.8692C>T | p.(Gln2898*) | g.31496468G>A | [13, 61] | DMD | 3 |
| 59 | c.8713C>T | p.(Arg2905*) | g.31496447G>A | [19, 55] | DMD | 34 |
| 59 | c.8728G>T | p.(Glu2910*) | g.31496432C>A | [25] | DMD | 1 |
| 59 | c.8745G>A | p.(Trp2915*) | g.31496415C>T | [27] | OTHER | 2 |
| 59 | c.8746G>T | p.(Glu2916*) | g.31496414C>A | [18] | DMD | 1 |
| 59 | c.8774G>A | p.(Trp2925*) | g.31496386C>T | [28] | ND | 2 |
| 59 | c.8775G>A | p.(Trp2925*) | g.31496385C>T | [19] | DMD | 1 |
| 59 | c.8776C>T | p.(Gln2926*) | g.31496384G>A | [89] | DMD | 1 |
| 59 | c.8791G>T | p.(Glu2931*) | g.31496369C>A | [1] | DMD | 1 |
| 59 | c.8800G>T | p.(Glu2934*) | g.31496360C>A | [90] | ND | 1 |
| 59 | c.8812G>T | p.(Glu2938*) | g.31496348C>A | [3] | DMD | 2 |
| 59 | c.8854C>T | p.(Gln2952*) | g.31496306G>A | [10] | DMD | 1 |
| 59 | c.8872G>T | p.(Gly2958*) | g.31496288C>A | [19] | DMD | 1 |
| 59 | c.8879G>A | p.(Trp2960*) | g.31496281C>T | [1] | DMD | 2 |
| 59 | c.8880G>A | p.(Trp2960*) | g.31496280C>T | [52] | ND | 1 |
| 59 | c.8914C>T | p.[Gln2972*, Val2955_Lys2979del] | g.31496246G>A | [28] | DMD | 4 |
| 59 | c.8929A>T | p.(Lys2977*) | g.31496231T>A | [1] | OTHER | 1 |
| 60 | c.8944C>T | p.(Arg2982*) | g.31462738G>A | [30] | DMD | 25 |
| 60 | c.8947G>T | p.(Gly2983*) | g.31462735C>A | [10] | DMD | 1 |
| 60 | c.9001C>T | p.(Gln3001*) | g.31462681G>A | [46] | DMD | 3 |
| 60 | c.9029C>G | p.(Ser3010*) | g.31462653G>C | [3] | DMD | 2 |
| 60 | c.9036T>A | p.(Tyr3012*) | g.31462646A>T | [13] | DMD | 1 |
| 60 | c.9071G>A | p.(Trp3024*) | g.31462611C>T | [1] | DMD | 1 |
| 60 | c.9072G>A | p.(Trp3024*) | g.31462610C>T | [21, 53, 55] | DMD | 3 |
| 60 | c.9082C>T | p.(Gln3028*) | g.31462600G>A | [19] | DMD | 2 |
| 61 | c.9100C>T | p.(Arg3034*) | g.31366736G>A | [19, 20, 37, 71] | DMD | 14 |
| 61 | c.9109C>T | p.(Gln3037*) | g.31366727G>A | [28] | DMD | 1 |
| 61 | c.9148C>T | p.(Gln3050*) | g.31366688G>A | [39] | DMD | 3 |
| 62 | c.9182G>A | p.(Trp3061*) | g.31341757C>T | [10] | DMD | 3 |
| 62 | c.9183G>A | p.(Trp3061*) | g.31341756C>T | [13] [54] | DMD | 3 |
| 62 | c.9197C>A | p.(Ser3066*) | g.31341742G>T | [11] | DMD | 2 |
| 62 | c.9216C>A | p.(Tyr3072*) | g.31341723G>T | [91] | DMD | 1 |
| 62 | c.9216C>G | p.(Tyr3072*) | g.31341723G>C | [18] | DMD | 2 |
| 63 | c.9249G>A | p.(Trp3083*) | g.31279109C>T | [20] | DMD | 2 |
| 63 | c.9276C>A | p.(Tyr3092*) | g.31279082G>T | [37] | ND | 1 |
| 63 | c.9284T>G | p.(Leu3095*) | g.31279074A>C | [21] | OTHER | 2 |
| 64 | c.9337C>T | p.(Arg3113*) | g.31241188G>A | [19] | DMD | 27 |
| 64 | c.9346C>T | p.(Gln3116*) | g.31241179G>A | [30] | DMD | 6 |
| 64 | c.9360C>A | p.(Cys3120*) | g.31241165G>T | [27] | DMD | 1 |
| 65 | c.9380C>G | p.(Ser3127*) | g.31227798G>C | [30] | DMD | 9 |
| 65 | c.9398T>A | p.(Leu3133*) | g.31227780A>T | [1] | DMD/BMD | 1 |
| 65 | c.9403C>T | p.(Gln3135*) | g.31227775G>A | [25] | DMD | 4 |
| 65 | c.9427C>T | p.(Gln3143*) | g.31227751G>A | [30] | DMD | 4 |
| 65 | c.9445C>T | p.(Gln3149*) | g.31227733G>A | [39] | DMD | 5 |
| 65 | c.9459T>A | p.(Cys3153*) | g.31227719A>T | [1] | ND | 1 |
| 65 | c.9461T>A | p.(Leu3154*) | g.31227717A>T | [28] | DMD | 2 |
| 65 | c.9474T>G | p.(Tyr3158*) | g.31227704A>C | [33] | ND | 1 |
| 65 | c.9522C>A | p.(Cys3174*) | g.31227656G>T | [21] | DMD | 4 |
| 65 | c.9542G>A | p.(Trp3181*) | g.31227636C>T | [13] | DMD | 2 |
| 66 | c.9568C>T | p.[Arg3190*, Gly3189Profs*13] | g.31224780G>A | [49] | DMD | 47 |
| 66 | c.9621T>A | p.(Cys3207*) | g.31224727A>T | [16] | ND | 1 |
| 66 | c.9640A>T | p.(Lys3214*) | g.31224708T>A | [1] | DMD | 1 |
| 67 | c.9651C>A | p.(Tyr3217*) | g.31222234G>T | [1] | OTHER | 1 |
| 67 | c.9661C>T | p.(Gln3231*) | g.31222224G>A | [37] | ND | 1 |
| 67 | c.9691C>T | p.(Gln3231*) | g.31222194G>A | [25, 91] | DMD | 2 |
| 67 | c.9739C>T | p.(Gln3247*) | g.31222146G>A | [15] | DMD | 3 |
| 67 | c.9748G>T | p.(Glu3250*) | g.31222137C>A | [12] | DMD | 1 |
| 68 | c.9829G>T | p.(Glu3277*) | g.31201000C>A | [52] | ND | 2 |
| 68 | c.9851G>A | p.(Trp3284*) | g.31200978C>T | [37] | DMD | 4 |
| 68 | c.9862G>T | p.(Glu3288*) | g.31200967C>A | [28] | DMD/BMD | 1 |
| 68 | c.9882G>A | p.(Trp3294*) | g.31200947C>T | [77] | DMD/BMD | 2 |
| 68 | c.9898A>T | p.(Arg3300*) | g.31200931T>A | [20] | DMD | 1 |
| 68 | c.9913G>T | p.(Glu3305*) | g.31200916C>A | [18] | DMD | 1 |
| 68 | c.9928C>T | p.(Gln3310*) | g.31200901G>A | [13, 19, 46] | DMD | 5 |
| 68 | c.9948C>A | p.(Cys3316*) | g.31200881G>T | [13] | DMD | 1 |
| 68 | c.9952G>T | p.(Glu3318*) | g.31200877C>A | [19] | DMD | 1 |
| 69 | c.9978C>G | p.(Tyr3326*) | g.31198595G>C | [19] | DMD | 1 |
| 69 | c.10002T>G | p.(Tyr3334*) | g.31198571A>C | [19] | DMD/BMD | 1 |
| 69 | c.10012C>T | p.(Gln3338*) | g.31198561G>A | [19] | DMD | 1 |
| 69 | c.10033C>T | p.(Arg3345*) | g.31198540G>A | [13, 20, 38, 53] | DMD | 19 |
| 69 | c.10062T>A | p.(Tyr3354*) | g.31198511A>T | [16] | ND | 1 |
| 69 | c.10072G>T | p.(Glu3358*) | g.31198501C>A | [12] | DMD | 1 |
| 69 | c.10077T>A | p.(Tyr3359*) | g.31198496A>T | [28] | DMD | 1 |
| 70 | c.10094C>G | p.(Ser3365*) | g.31196915G>C | [1] | DMD/BMD | 1 |
| 70 | c.10096G>T | p.(Gly3366*) | g.31196913C>A | [13] | DMD | 1 |
| 70 | c.10108C>T | p.(Arg3370*) | g.31196901G>A | [10] | DMD | 44 |
| 70 | c.10135A>T | p.(Lys3379*) | g.31196874T>A | [25, 91] | DMD | 2 |
| 70 | c.10141C>T | p.(Arg3381*) | g.31196868G>A | [10] | DMD | 55 |
| 70 | c.10147A>T | p.(Lys3383*) | g.31196862T>A | [1] | DMD | 1 |
| 70 | c.10171C>T | p.(Arg3391*) | g.31196838G>A | [92] | DMD | 46 |
| 70 | c.10192C>T | p.(Gln3398*) | g.31196817G>A | [1] | DMD/BMD | 1 |
| 70 | c.10202T>G | p.(Leu3401*) | g.31196807A>C | [33] | DMD | 2 |
| 70 | c.10219G>T | p.(Glu3407*) | g.31196790C>A | [71] | ND | 2 |
| 71 | c.10247G>A | p.(Trp3416*) | g.31196064C>T | [93] | OTHER | 1 |
| 72 | c.10279C>T | p.[Gln3427*, Pro3422_Arg3443del] | g.31191705G>A | [25] | BMD | 7 |
| 72 | c.10320T>A | p.(Tyr3440*) | g.31191664A>T | [13] | DMD/BMD | 3 |
| 73 | c.10362T>A | p.(Tyr3454*) | g.31190497A>T | [1] | BMD | 1 |
| 73 | c.10387G>T | p.(Glu3463*) | g.31190472C>A | [1] | ND | 1 |
| 74 | c.10412T>G | p.(Leu3471*) | g.31187701A>C | [74] | DMD | 1 |
| 74 | c.10412T>A | p.(Leu3471*) | g.31187701A>T | [74] | BMD | 2 |
| 74 | c.10429C>T | p.(Gln3477*) | g.31187684G>A | [1] | BMD | 1 |
| 74 | c.10477C>T | p.(Gln3493*) | g.31187636G>A | [84] | DMD/BMD | 5 |
| 74 | c.10493T>A | p.(Leu3498*) | g.31187620A>T | [1] | DMD | 1 |
| 74 | c.10495G>T | p.(Glu3499*) | g.31187618C>A | [13] | DMD | 2 |
| 74 | c.10504G>T | p.(Glu3502*) | g.31187609C>A | [34] | DMD | 1 |
| 74 | c.10519G>T | p.(Glu3507*) | g.31187594C>A | [16] | ND | 1 |
| 74 | c.10543G>T | p.[Glu3515*, Ile3465_Arg3518delinsMet] | g.31187570C>A | [15] | BMD | 1 |
| 74 | c.10546G>T | p.(Glu3516*) | g.31187567C>A | [42, 53] | DMD/BMD | 3 |
| 75 | c.10572T>A | p.(Tyr3524*) | g.31165617A>T | [1] | DMD | 1 |
| 75 | c.10588C>T | p.(Gln3530*) | g.31165601G>A | [1] | DMD | 3 |
| 75 | c.10651C>T | p.(Gln3551*) | g.31165538G>A | [13] | DMD | 3 |
| 75 | c.10759G>T | p.(Glu3587*) | g.31165430C>A | [1] | BMD | 1 |
| 75 | c.10765C>T | p.(Gln3589*) | g.31165424G>A | [1] | ND | 1 |
| 75 | c.10769T>A | p.(Leu3590*) | g.31165420A>T | [1] | OTHER | 1 |
| 75 | c.10783C>T | p.(Gln3595*) | g.31165406G>A | [71] | DMD/BMD | 3 |
| 75 | c.10792G>T | p.(Glu3598*) | g.31165397C>A | [13] | BMD | 1 |
| 76 | c.10801C>T | p.(Gln3601*) | g.31164528G>A | [19] | DMD | 3 |
| 76 | c.10855C>T | p.(Gln3619*) | g.31164474G>A | [94] | DMD/BMD | 1 |
| 76 | c.10873C>T | p.(Gln3625*) | g.31164456G>A | [18] | BMD | 1 |
| 76 | c.10888C>T | p.(Arg3630*) | g.31164441G>A | [19] | BMD | 1 |
| 76 | c.10903C>T | p.(Gln3635*) | g.31164426G>A | [30] | DMD | 3 |
| 76 | c.10910C>A | p.(Ser3637*) | g.31164419G>T | [25, 91] | BMD | 4 |

**References**

1. 1. Leiden Open Variation Database. LOVD 2020, April 14 [Available from: <https://databases.lovd.nl/shared/genes/DMD>.

2. Gurvich OL, Maiti B, Weiss RB, Aggarwal G, Howard MT, Flanigan KM. DMD exon 1 truncating point mutations: amelioration of phenotype by alternative translation initiation in exon 6. Hum Mutat. 2009;30(4):633-40.

3. Ma P, Zhang S, Zhang H, Fang S, Dong Y, Zhang Y, et al. Comprehensive genetic characteristics of dystrophinopathies in China. Orphanet J Rare Dis. 2018;13(1):109.

4. Vieitez I, Gallano P, Gonzalez-Quereda L, Borrego S, Marcos I, Millan JM, et al. Mutational spectrum of Duchenne muscular dystrophy in Spain: Study of 284 cases. Neurologia. 2017;32(6):377-85.

5. Witting N, Duno M, Vissing J. Becker muscular dystrophy with widespread muscle hypertrophy and a non-sense mutation of exon 2. Neuromuscul Disord. 2013;23(1):25-8.

6. Oitani Y, Ishiyama A, Kosuga M, Iwasawa K, Ogata A, Tanaka F, et al. Interpretation of acid alpha-glucosidase activity in creatine kinase elevation: A case of Becker muscular dystrophy. Brain Dev. 2018;40(9):837-40.

7. Bai Y, Li S, Zong YN, Li XL, Zhao ZH, Kong XD. [Mutation screening of 433 families with Duchenne/Becker muscular dystrophy]. Zhonghua Yi Xue Za Zhi. 2016;96(16):1261-9.

8. Garcia-Planells J, Torres-Puente M, Vilchez JJ, Perez-Alonso M. Novel human pathological mutations. Gene symbol: DMD. Disease: muscular dystrophy, Duchenne. Hum Genet. 2009;126(2):338.

9. Zhong J, Xie Y, Bhandari V, Chen G, Dang Y, Liao H, et al. Clinical and genetic characteristics of female dystrophinopathy carriers. Mol Med Rep. 2019;19(4):3035-44.

10. Sedlackova J, Vondracek P, Hermanova M, Zamecnik J, Hruba Z, Haberlova J, et al. Point mutations in Czech DMD/BMD patients and their phenotypic outcome. Neuromuscul Disord. 2009;19(11):749-53.

11. Roberts RG, Gardner RJ, Bobrow M. Searching for the 1 in 2,400,000: a review of dystrophin gene point mutations. Hum Mutat. 1994;4(1):1-11.

12. Dolinsky LC, de Moura-Neto RS, Falcao-Conceicao DN. DGGE analysis as a tool to identify point mutations, de novo mutations and carriers of the dystrophin gene. Neuromuscul Disord. 2002;12(9):845-8.

13. Okubo M, Goto K, Komaki H, Nakamura H, Mori-Yoshimura M, Hayashi YK, et al. Comprehensive analysis for genetic diagnosis of Dystrophinopathies in Japan. Orphanet J Rare Dis. 2017;12(1):149.

14. Sitnik R, Campiotto S, Vainzof M, Pavanello RC, Takata RI, Zatz M, et al. Novel point mutations in the dystrophin gene. Hum Mutat. 1997;10(3):217-22.

15. Hofstra RM, Mulder IM, Vossen R, de Koning-Gans PA, Kraak M, Ginjaar IB, et al. DGGE-based whole-gene mutation scanning of the dystrophin gene in Duchenne and Becker muscular dystrophy patients. Hum Mutat. 2004;23(1):57-66.

16. NCBI. ClinVar 2020, April 14 [Available from: <https://www.ncbi.nlm.nih.gov/clinvar/>.

17. Nigro V, Nigro G, Esposito MG, Comi LI, Molinari AM, Puca GA, et al. Novel small mutations along the DMD/BMD gene associated with different phenotypes. Hum Mol Genet. 1994;3(10):1907-8.

18. Takeshima Y, Yagi M, Okizuka Y, Awano H, Zhang Z, Yamauchi Y, et al. Mutation spectrum of the dystrophin gene in 442 Duchenne/Becker muscular dystrophy cases from one Japanese referral center. J Hum Genet. 2010;55(6):379-88.

19. Flanigan KM, Dunn DM, von Niederhausern A, Soltanzadeh P, Gappmaier E, Howard MT, et al. Mutational spectrum of DMD mutations in dystrophinopathy patients: application of modern diagnostic techniques to a large cohort. Hum Mutat. 2009;30(12):1657-66.

20. Cho A, Seong MW, Lim BC, Lee HJ, Byeon JH, Kim SS, et al. Consecutive analysis of mutation spectrum in the dystrophin gene of 507 Korean boys with Duchenne/Becker muscular dystrophy in a single center. Muscle Nerve. 2017;55(5):727-34.

21. Wang D, Gao M, Zhang K, Jin R, Lv Y, Liu Y, et al. Molecular Genetics Analysis of 70 Chinese Families With Muscular Dystrophy Using Multiplex Ligation-Dependent Probe Amplification and Next-Generation Sequencing. Front Pharmacol. 2019;10:814.

22. Bonnal RJ, Severgnini M, Castaldi A, Bordoni R, Iacono M, Trimarco A, et al. Reliable resequencing of the human dystrophin locus by universal long polymerase chain reaction and massive pyrosequencing. Anal Biochem. 2010;406(2):176-84.

23. Guo R, Zhu G, Zhu H, Ma R, Peng Y, Liang D, et al. DMD mutation spectrum analysis in 613 Chinese patients with dystrophinopathy. J Hum Genet. 2015;60(8):435-42.

24. Yang J, Li SY, Li YQ, Cao JQ, Feng SW, Wang YY, et al. MLPA-based genotype-phenotype analysis in 1053 Chinese patients with DMD/BMD. BMC Med Genet. 2013;14:29.

25. Deburgrave N, Daoud F, Llense S, Barbot JC, Recan D, Peccate C, et al. Protein- and mRNA-based phenotype-genotype correlations in DMD/BMD with point mutations and molecular basis for BMD with nonsense and frameshift mutations in the DMD gene. Hum Mutat. 2007;28(2):183-95.

26. Toksoy G, Durmus H, Aghayev A, Bagirova G, Sevinc Rustemoglu B, Basaran S, et al. Mutation spectrum of 260 dystrophinopathy patients from Turkey and important highlights for genetic counseling. Neuromuscul Disord. 2019;29(8):601-13.

27. Lo IF, Lai KK, Tong TM, Lam ST. A different spectrum of DMD gene mutations in local Chinese patients with Duchenne/Becker muscular dystrophy. Chin Med J (Engl). 2006;119(13):1079-87.

28. Taylor PJ, Maroulis S, Mullan GL, Pedersen RL, Baumli A, Elakis G, et al. Measurement of the clinical utility of a combined mutation detection protocol in carriers of Duchenne and Becker muscular dystrophy. J Med Genet. 2007;44(6):368-72.

29. Santos R, Goncalves A, Oliveira J, Vieira E, Vieira JP, Evangelista T, et al. New variants, challenges and pitfalls in DMD genotyping: implications in diagnosis, prognosis and therapy. J Hum Genet. 2014;59(8):454-64.

30. Mendell JR, Buzin CH, Feng J, Yan J, Serrano C, Sangani DS, et al. Diagnosis of Duchenne dystrophy by enhanced detection of small mutations. Neurology. 2001;57(4):645-50.

31. Karbassi I, Maston GA, Love A, DiVincenzo C, Braastad CD, Elzinga CD, et al. A Standardized DNA Variant Scoring System for Pathogenicity Assessments in Mendelian Disorders. Hum Mutat. 2016;37(1):127-34.

32. Shen BC, Zhang C, Chen SL, Sun XF, Li SY, Yao XL, et al. [Identification of disease-causing point mutations in DMD patients' dystrophin gene without large deletions/duplications]. Zhonghua Yi Xue Yi Chuan Xue Za Zhi. 2006;23(4):392-6.

33. Neri M, Rossi R, Trabanelli C, Mauro A, Selvatici R, Falzarano MS, et al. The Genetic Landscape of Dystrophin Mutations in Italy: A Nationwide Study. Front Genet. 2020;11:131.

34. Prior TW, Bridgeman SJ. Experience and strategy for the molecular testing of Duchenne muscular dystrophy. J Mol Diagn. 2005;7(3):317-26.

35. Nigro V, Politano L, Nigro G, Romano SC, Molinari AM, Puca GA. Detection of a nonsense mutation in the dystrophin gene by multiple SSCP. Hum Mol Genet. 1992;1(7):517-20.

36. Cunniff C, Andrews J, Meaney FJ, Mathews KD, Matthews D, Ciafaloni E, et al. Mutation analysis in a population-based cohort of boys with Duchenne or Becker muscular dystrophy. J Child Neurol. 2009;24(4):425-30.

37. Mah JK, Selby K, Campbell C, Nadeau A, Tarnopolsky M, McCormick A, et al. A population-based study of dystrophin mutations in Canada. Can J Neurol Sci. 2011;38(3):465-74.

38. Okubo M, Noguchi S, Hayashi S, Nakamura H, Komaki H, Matsuo M, et al. Exon skipping induced by nonsense/frameshift mutations in DMD gene results in Becker muscular dystrophy. Hum Genet. 2020;139(2):247-55.

39. Tuffery-Giraud S, Saquet C, Chambert S, Echenne B, Marie Cuisset J, Rivier F, et al. The role of muscle biopsy in analysis of the dystrophin gene in Duchenne muscular dystrophy: experience of a national referral centre. Neuromuscul Disord. 2004;14(10):650-8.

40. Lim BC, Lee S, Shin JY, Kim JI, Hwang H, Kim KJ, et al. Genetic diagnosis of Duchenne and Becker muscular dystrophy using next-generation sequencing technology: comprehensive mutational search in a single platform. J Med Genet. 2011;48(11):731-6.

41. Altarescu G, Eldar-Geva T, Varshower I, Brooks B, Haran EZ, Margalioth EJ, et al. Real-time reverse linkage using polar body analysis for preimplantation genetic diagnosis in female carriers of de novo mutations. Hum Reprod. 2009;24(12):3225-9.

42. Hwa HL, Chang YY, Huang CH, Chen CH, Kao YS, Jong YJ, et al. Small mutations of the DMD gene in Taiwanese families. J Formos Med Assoc. 2008;107(6):463-9.

43. Yang YM, Yan K, Liu B, Chen M, Wang LY, Huang YZ, et al. Comprehensive genetic diagnosis of patients with Duchenne/Becker muscular dystrophy (DMD/BMD) and pathogenicity analysis of splice site variants in the DMD gene. J Zhejiang Univ Sci B. 2019;20(9):753-65.

44. Kong X, Zhong X, Liu L, Cui S, Yang Y, Kong L. Genetic analysis of 1051 Chinese families with Duchenne/Becker Muscular Dystrophy. BMC Med Genet. 2019;20(1):139.

45. Whittock NV, Roberts RG, Mathew CG, Abbs SJ. Dystrophin point mutation screening using a multiplexed protein truncation test. Genet Test. 1997;1(2):115-23.

46. Buzin CH, Feng J, Yan J, Scaringe W, Liu Q, den Dunnen J, et al. Mutation rates in the dystrophin gene: a hotspot of mutation at a CpG dinucleotide. Hum Mutat. 2005;25(2):177-88.

47. Almomani R, van der Stoep N, Bakker E, den Dunnen JT, Breuning MH, Ginjaar IB. Rapid and cost effective detection of small mutations in the DMD gene by high resolution melting curve analysis. Neuromuscul Disord. 2009;19(6):383-90.

48. Hamed SA, Hoffman EP. Automated sequence screening of the entire dystrophin cDNA in Duchenne dystrophy: point mutation detection. Am J Med Genet B Neuropsychiatr Genet. 2006;141B(1):44-50.

49. Wang H, Xu Y, Liu X, Wang L, Jiang W, Xiao B, et al. Prenatal diagnosis of Duchenne muscular dystrophy in 131 Chinese families with dystrophinopathy. Prenat Diagn. 2017;37(4):356-64.

50. Luce LN, Carcione M, Mazzanti C, Ferrer M, Szijan I, Giliberto F. Small mutation screening in the DMD gene by whole exome sequencing of an argentine Duchenne/Becker muscular dystrophies cohort. Neuromuscul Disord. 2018;28(12):986-95.

51. Spitali P, Zaharieva I, Bohringer S, Hiller M, Chaouch A, Roos A, et al. TCTEX1D1 is a genetic modifier of disease progression in Duchenne muscular dystrophy. Eur J Hum Genet. 2020.

52. Torella A, Trimarco A, Blanco Fdel V, Cuomo A, Aurino S, Piluso G, et al. One hundred twenty-one dystrophin point mutations detected from stored DNA samples by combinatorial denaturing high-performance liquid chromatography. J Mol Diagn. 2010;12(1):65-73.

53. Xu Y, Li Y, Song T, Guo F, Zheng J, Xu H, et al. A retrospective analysis of 237 Chinese families with Duchenne muscular dystrophy history and strategies of prenatal diagnosis. J Clin Lab Anal. 2018;32(7):e22445.

54. Spitali P, Rimessi P, Fabris M, Perrone D, Falzarano S, Bovolenta M, et al. Exon skipping-mediated dystrophin reading frame restoration for small mutations. Hum Mutat. 2009;30(11):1527-34.

55. Prior TW, Bartolo C, Pearl DK, Papp AC, Snyder PJ, Sedra MS, et al. Spectrum of small mutations in the dystrophin coding region. Am J Hum Genet. 1995;57(1):22-33.

56. Tomar S, Moorthy V, Sethi R, Chai J, Low PS, Hong STK, et al. Mutational spectrum of dystrophinopathies in Singapore: Insights for genetic diagnosis and precision therapy. Am J Med Genet C Semin Med Genet. 2019;181(2):230-44.

57. Vogiatzakis N, Kekou K, Sophocleous C, Kitsiou S, Mavrou A, Bakoula C, et al. Screening human genes for small alterations performing an enzymatic cleavage mismatched analysis (ECMA) protocol. Mol Biotechnol. 2007;37(3):212-9.

58. Roberts RG, Bobrow M, Bentley DR. Point mutations in the dystrophin gene. Proc Natl Acad Sci U S A. 1992;89(6):2331-5.

59. Wei X, Dai Y, Yu P, Qu N, Lan Z, Hong X, et al. Targeted next-generation sequencing as a comprehensive test for patients with and female carriers of DMD/BMD: a multi-population diagnostic study. Eur J Hum Genet. 2014;22(1):110-8.

60. Alcantara-Ortigoza MA, Reyna-Fabian ME, Gonzalez-Del Angel A, Estandia-Ortega B, Bermudez-Lopez C, Cruz-Miranda GM, et al. Predominance of Dystrophinopathy Genotypes in Mexican Male Patients Presenting as Muscular Dystrophy with A Normal Multiplex Polymerase Chain Reaction DMD Gene Result: A Study Including Targeted Next-Generation Sequencing. Genes (Basel). 2019;10(11).

61. Tallapaka K, Ranganath P, Ramachandran A, Uppin MS, Perala S, Aggarwal S, et al. Molecular and Histopathological Characterization of Patients Presenting with the Duchenne Muscular Dystrophy Phenotype in a Tertiary Care Center in Southern India. Indian Pediatr. 2019;56(7):556-9.

62. Li S, Bai Y, Zhao Z, Kong X. [Mutation analysis of 81 cases with Duchenne/Becker muscular dystrophy]. Zhonghua Yi Xue Yi Chuan Xue Za Zhi. 2016;33(6):762-7.

63. Saillour Y, Cossee M, Leturcq F, Vasson A, Beugnet C, Poirier K, et al. Detection of exonic copy-number changes using a highly efficient oligonucleotide-based comparative genomic hybridization-array method. Hum Mutat. 2008;29(9):1083-90.

64. Dasouki M, Barohn R. Gene symbol: DMD. Disease: Muscular dystrophy, Duchenne. Hum Genet. 2008;124(3):321.

65. Chen C, Ma H, Zhang F, Chen L, Xing X, Wang S, et al. Screening of Duchenne muscular dystrophy (DMD) mutations and investigating its mutational mechanism in Chinese patients. PLoS One. 2014;9(9):e108038.

66. Aartsma-Rus A, Van Deutekom JC, Fokkema IF, Van Ommen GJ, Den Dunnen JT. Entries in the Leiden Duchenne muscular dystrophy mutation database: an overview of mutation types and paradoxical cases that confirm the reading-frame rule. Muscle Nerve. 2006;34(2):135-44.

67. Adachi K, Yagi M, Ito T, Takeshima Y, Nishio H, Wada H, et al. [Dystrophin gene analysis on 76 families with dystrophinopathy]. No To Hattatsu. 2002;34(5):391-7.

68. Rani AQ, Sasongko TH, Sulong S, Bunyan D, Salmi AR, Zilfalil BA, et al. Mutation spectrum of dystrophin gene in malaysian patients with Duchenne/Becker muscular dystrophy. J Neurogenet. 2013;27(1-2):11-5.

69. de Almeida PAD, Machado-Costa MC, Manzoli GN, Ferreira LS, Rodrigues MCS, Bueno LSM, et al. Genetic profile of Brazilian patients with dystrophinopathies. Clin Genet. 2017;92(2):199-203.

70. Ashton E, Deans Z, Yau SC, Abbs S. A novel and rapid mutation screening approach facilitates prenatal diagnosis. Prenat Diagn. 2005;25(5):425-6.

71. Magri F, Del Bo R, D'Angelo MG, Govoni A, Ghezzi S, Gandossini S, et al. Clinical and molecular characterization of a cohort of patients with novel nucleotide alterations of the Dystrophin gene detected by direct sequencing. BMC Med Genet. 2011;12:37.

72. Lopez-Hernandez LB, Gomez-Diaz B, Luna-Angulo AB, Anaya-Segura M, Bunyan DJ, Zuniga-Guzman C, et al. Comparison of mutation profiles in the Duchenne muscular dystrophy gene among populations: implications for potential molecular therapies. Int J Mol Sci. 2015;16(3):5334-46.

73. Piko H, Vancso V, Nagy B, Ban Z, Herczegfalvi A, Karcagi V. Dystrophin gene analysis in Hungarian Duchenne/Becker muscular dystrophy families - detection of carrier status in symptomatic and asymptomatic female relatives. Neuromuscul Disord. 2009;19(2):108-12.

74. Tuffery-Giraud S, Saquet C, Thorel D, Disset A, Rivier F, Malcolm S, et al. Mutation spectrum leading to an attenuated phenotype in dystrophinopathies. Eur J Hum Genet. 2005;13(12):1254-60.

75. Ebrahimzadeh-Vesal R, Teymoori A, Azimi-Nezhad M, Hosseini FS. Next Generation Sequencing approach to molecular diagnosis of Duchenne muscular dystrophy; identification of a novel mutation. Gene. 2018;644:1-3.

76. Lee BL, Nam SH, Lee JH, Ki CS, Lee M, Lee J. Genetic analysis of dystrophin gene for affected male and female carriers with Duchenne/Becker muscular dystrophy in Korea. J Korean Med Sci. 2012;27(3):274-80.

77. Hamed S, Sutherland-Smith A, Gorospe J, Kendrick-Jones J, Hoffman E. DNA sequence analysis for structure/function and mutation studies in Becker muscular dystrophy. Clin Genet. 2005;68(1):69-79.

78. Janssen B, Hartmann C, Scholz V, Jauch A, Zschocke J. MLPA analysis for the detection of deletions, duplications and complex rearrangements in the dystrophin gene: potential and pitfalls. Neurogenetics. 2005;6(1):29-35.

79. Kalman L, Leonard J, Gerry N, Tarleton J, Bridges C, Gastier-Foster JM, et al. Quality assurance for Duchenne and Becker muscular dystrophy genetic testing: development of a genomic DNA reference material panel. J Mol Diagn. 2011;13(2):167-74.

80. Zhang Y, Yang W, Wen G, Wu Y, Jing Z, Li D, et al. Application whole exome sequencing for the clinical molecular diagnosis of patients with Duchenne muscular dystrophy; identification of four novel nonsense mutations in four unrelated Chinese DMD patients. Mol Genet Genomic Med. 2019;7(5):e622.

81. Marquis-Nicholson R, Lai D, Lan CC, Love JM, Love DR. A Streamlined Protocol for Molecular Testing of the DMD Gene within a Diagnostic Laboratory: A Combination of Array Comparative Genomic Hybridization and Bidirectional Sequence Analysis. ISRN Neurol. 2013;2013:908317.

82. Kohli S, Saxena R, Thomas E, Singh J, Verma IC. Gene changes in Duchenne muscular dystrophy: comparison of multiplex PCR and multiplex ligation-dependent probe amplification techniques. Neurol India. 2010;58(6):852-6.

83. Clemens PR, Ward PA, Caskey CT, Bulman DE, Fenwick RG. Premature chain termination mutation causing Duchenne muscular dystrophy. Neurology. 1992;42(9):1775-82.

84. Nallamilli BRR, Chakravorty S, Kesari A, Tanner A, Ankala A, Schneider T, et al. Genetic landscape and novel disease mechanisms from a large LGMD cohort of 4656 patients. Ann Clin Transl Neurol. 2018;5(12):1574-87.

85. Yu H, Chen YC, Liu GL, Wu ZY. A De novo Mutation in Dystrophin Causing Muscular Dystrophy in a Female Patient. Chin Med J (Engl). 2017;130(19):2273-8.

86. Ribeiro J, Rebelo O, Fernandez-Marmiesse A, Negrao L. Novel mosaic mutation in the dystrophin gene causing distal asymmetric muscle weakness of the upper limbs and dilated cardiomyopathy. Acta Myol. 2018;37(2):117-20.

87. Tuffery-Giraud S, Chambert S, Demaille J, Claustres M. Point mutations in the dystrophin gene: evidence for frequent use of cryptic splice sites as a result of splicing defects. Hum Mutat. 1999;14(5):359-68.

88. Chen YN, Zhou X, Jin CL, Xu Y, Lin CK, Cao LH, et al. [Detection of new mutations in the dystrophin gene by denaturing high-performance liquid chromatography]. Zhonghua Er Ke Za Zhi. 2007;45(6):413-6.

89. Todorova A, Todorov T, Georgieva B, Lukova M, Guergueltcheva V, Kremensky I, et al. MLPA analysis/complete sequencing of the DMD gene in a group of Bulgarian Duchenne/Becker muscular dystrophy patients. Neuromuscul Disord. 2008;18(8):667-70.

90. Suh MR, Lee KA, Kim EY, Jung J, Choi WA, Kang SW. Multiplex Ligation-Dependent Probe Amplification in X-linked Recessive Muscular Dystrophy in Korean Subjects. Yonsei Med J. 2017;58(3):613-8.

91. Daoud F, Angeard N, Demerre B, Martie I, Benyaou R, Leturcq F, et al. Analysis of Dp71 contribution in the severity of mental retardation through comparison of Duchenne and Becker patients differing by mutation consequences on Dp71 expression. Hum Mol Genet. 2009;18(20):3779-94.

92. Barbieri AM, Soriani N, Ferlini A, Michelato A, Ferrari M, Carrera P. Seven novel additional small mutations and a new alternative splicing in the human dystrophin gene detected by heteroduplex analysis and restricted RT-PCR heteroduplex analysis of illegitimate transcripts. Eur J Hum Genet. 1996;4(3):183-7.

93. Richards S, Aziz N, Bale S, Bick D, Das S, Gastier-Foster J, et al. Standards and guidelines for the interpretation of sequence variants: a joint consensus recommendation of the American College of Medical Genetics and Genomics and the Association for Molecular Pathology. Genet Med. 2015;17(5):405-24.

94. Chen WJ, Lin QF, Zhang QJ, He J, Liu XY, Lin MT, et al. Molecular analysis of the dystrophin gene in 407 Chinese patients with Duchenne/Becker muscular dystrophy by the combination of multiplex ligation-dependent probe amplification and Sanger sequencing. Clin Chim Acta. 2013;423:35-8.
